# Glycolysis-related gene expression profiling serves as a novel prognosis risk predictor for human hepatocellular carcinoma

**DOI:** 10.1038/s41598-021-98381-2

**Published:** 2021-09-23

**Authors:** Lingyu Zhang, Yu Li, Yibei Dai, Danhua Wang, Xuchu Wang, Ying Cao, Weiwei Liu, Zhihua Tao

**Affiliations:** 1 Department of Laboratory Medicine, The Second Affiliated Hospital of Zhejiang University School of Medicine, 88 Jiefang Road, Hangzhou, 310009 Zhejiang People’s Republic of China; 2 Department of Biochemistry and Molecular Biology, Bengbu Medical College, Anhui, 233030 People’s Republic of China

**Keywords:** Cancer, Computational biology and bioinformatics, Genetics, Immunology, Molecular biology

## Abstract

Metabolic pattern reconstruction is an important factor in tumor progression. Metabolism of tumor cells is characterized by abnormal increase in anaerobic glycolysis, regardless of high oxygen concentration, resulting in a significant accumulation of energy from glucose sources. These changes promotes rapid cell proliferation and tumor growth, which is further referenced a process known as the Warburg effect. The current study reconstructed the metabolic pattern in progression of cancer to identify genetic changes specific in cancer cells. A total of 12 common types of solid tumors were included in the current study. Gene set enrichment analysis (GSEA) was performed to analyze 9 glycolysis-related gene sets, which are implicated in the glycolysis process. Univariate and multivariate analyses were used to identify independent prognostic variables for construction of a nomogram based on clinicopathological characteristics and a glycolysis-related gene prognostic index (GRGPI). The prognostic model based on glycolysis genes showed high area under the curve (AUC) in LIHC (Liver hepatocellular carcinoma). The findings of the current study showed that 8 genes (AURKA, CDK1, CENPA, DEPDC1, HMMR, KIF20A, PFKFB4, STMN1) were correlated with overall survival (OS) and recurrence-free survival (RFS). Further analysis showed that the prediction model accurately distinguished between high- and low-risk cancer patients among patients in different clusters in LIHC. A nomogram with a well-fitted calibration curve based on gene expression profiles and clinical characteristics showed good discrimination based on internal and external cohorts. These findings indicate that changes in expression level of metabolic genes implicated in glycolysis can contribute to reconstruction of tumor-related microenvironment.

## Introduction

Cells undergo changes in energy metabolism patterns for biosynthesis, depending on cell function and availability of metabolites. In addition to oxidative phosphorylation of glucose, other metabolic pathways, including lipid, nucleotide, and amino acid metabolism can provide energy to meet the biosynthetic requirements for cell growth and proliferation^1,2^. Energy metabolism pattern of tumor cells shows significant differences, compared with oxidative phosphorylation (OXPHOS) in normal cells. Energy metabolism is reprogrammed in tumor cells, in a process known as Warburg effect to maintain survival and meet the high demand for synthesis of biological macromolecules^3–5^. Warburg effect represents change in glucose utilization by tumor cells from oxidative phosphorylation to glycolysis, which is now acknowledged as a major feature hallmark of tumors^6,7^. This change in energy metabolism is determined by complex factors, including pressure on tumor microenvironment and genetic changes^8–11^. Enhanced glycolysis of tumor cells is mainly promoted by increased expression or activity of key glycolysis enzymes^12^. Previous studies have explored agents that target tumors by inhibiting activity of key enzymes in the tumor glycolysis pathway. In addition, studies report that specific inhibition of glycolysis is associated with significant tumor suppression, and induces cell death. Glycolytic key enzymes such as hexokinase 2 (HK2), phosphofructosidase (PFK), and M2-type acetone kinase (PKM2) are tumor markers, and their expression and activity can affect tumor glycolysis, which in turn affects proliferation of tumor cells^13–17^. However, studies have not explored glycolytic-related factors in refining stratification and management of cancer patients. Early diagnosis and personalized treatment can effectively improve survival of cancer patients. Histopathology analysis can be used to predict prognosis and outcome of cancer patients. However, patients with the same pathology present with different prognoses owing to different molecular subtypes thus limiting use of histopathological characteristics^18,19^. Advances in high-throughput nucleotide sequencing technology in the recent years enables better understanding of the dynamic changes in tumor cells at the molecular level. A single gene cannot accurately predict the outcome of cancer patients. However, several biomarker combinations can improve sensitivity and specificity of patient outcomes. Multiple biomarkers that are highly correlated with survival and prognosis can identify high-risk patients, ameliorate poor prognosis of cancer patients, and can be used for development of effective intervention therapy.

Gene set enrichment analysis (GSEA) is used in genomic research to identify potential biological mechanisms implicated in disease. In the current study, a gene signature was developed through GSEA analysis, based on genes implicated in glycolytic metabolic pathways. Tumor glycolysis metabolic patterns of 12 cancer types (Bladder Urothelial Carcinoma, BLCA; Breast invasive carcinoma, BRCA; Colon adenocarcinoma, COAD; Head and Neck squamous cell carcinoma, HNSC; Kidney renal clear cell carcinoma, KIRC; Kidney renal papillary cell carcinoma; KIRP; Liver hepatocellular carcinoma; LIHC; Lung adenocarcinoma, LUAD; Lung squamous cell carcinoma, LUSC; Ovarian serous cystadenocarcinoma, OV; Prostate adenocarcinoma, PRAD; Thyroid carcinoma, THCA), were explored through a comprehensive analysis of genome and transcriptome profiles of TCGA dataset. A GRGPI signature was developed for LIHC and multiple risk characteristics that can effectively predict prognosis of patients were determined. The findings showed that glycolysis-related risk characteristics can be used to identify patients with poor outcomes in high-risk group. In addition, Cox multivariate hazard ratio analysis showed that the risk score performed better compared with other clinical variable in evaluating patient prognosis.

## Materials and methods

### Gene expression profiles and patient clinical information

Transcriptome expression profiles were obtained from multiple data repositories, including The Cancer Genome Atlas Program (TCGA, https://portal.gdc.cancer.gov/), International Cancer Genome Consortium (ICGC, http://www.icgc.org) database, and Gene Expression Omnibus (GEO, http://www.ncbi.nlm.nih.gov/geo/) database. Datasets with insufficient sample size (< 200) or missing clinical information were excluded. The raw counts were transformed into transcripts per kilobase million (TPM) values for subsequent analysis.

### Gene set enrichment analysis

Molecular Signatures Database (MSigDB) was used to identify gene sets and specific biological processes that are significantly differentially expressed in different groups. Analysis using MSigDB resulted in a statistically significant improvement in connectivity between data expression pattern and biological processes, ignoring the clear differential gene threshold^20^. A total of 9 gene sets associated with glycolysis processes including glycolytic fermentation, glycolytic process, hallmark glycolysis, glycolysis gluconeogenesis, module 306, reactome glycolysis, and reactome regulation of glycolysis by fructose-2-6 bisphosphate metabolism, were retrieved from MsigDB. Permutations were performed 1000 times for each gene set. Normalized enrichment scores (NES) and FDR values were used to explore enriched pathways in each phenotype. GSEA was performed to explore differences in glycolysis-related gene sets between tumor tissues and matched normal tissues. P value and FDR value at < 0.05 were set as the threshold.

### Construction of risk prediction model and statistical analysis

Univariate Cox regression models were constructed to explore the statistical relationships between mRNA expression levels and RFS or OS. A linear regression model with a stepwise forward method was used to predict significant variations between variables, with the beta value (β) from univariate Cox regression analysis as the weighting factor^21^. Multivariable logistic regression analysis was performed after the LASSO (least absolute shrinkage and selection operator) analysis, which simultaneously selects the variables and penalizes the model coefficients for overoptimism^22^. Multivariate analysis was performed using the Cox proportional hazards (Cox-PH) model to identify independent predictors of survival. Covariates with a P value < 0.05 were used for subsequent risk prediction model construction based on multivariate analyses results. Standardized risk score was calculated using the formula shown below:

$$Risk\;Score = \mathop \sum \limits_{i = 1}^{n} \beta_{i} Expression\;(G_{i} ).$$

Patients with complete clinicopathological characteristics were divided into a high- and a low-risk group, based on the median value of the risk score. Kaplan–Meier curves wee generated to compare differences in survival probability in low- and high-risk groups. Log-rank test P < 0.05 was conducted to explore significance of survival time differences. All these analyses were performed packages in R version 3.6.1^23^. P value less than 0.05 denoted statistical significance.

### Immunohistochemistry (IHC) analysis

Immunohistochemical slides and relative clinical pathology information were retrieved from the Human Protein Atlas database (Ensembl version: 92.38) (HPA, https://www.proteinatlas.org/)^24^. Immunohistochemical staining results were evaluated by two independent pathologists, based on the integrated index by multiplying the intensity by the proportion of immunopositive cells of interest.

### Weighted gene co‐expression network analysis

To explore transcriptomic differences between HCC subgroups, weighted gene co-expression analysis was performed based on the unique characteristics of the subgroups to identify potential functional modules that can characterize biological functions of each subgroup. The optimal soft threshold parameter β (β = 7) was used to construct a scale-free co-expression network. Subsequently, genes with the same expression pattern based on Pearson's coefficient were concentrated into specific gene modules. The top 2 modules that had the strongest association with subgroups were selected for further analysis. GO and KEGG pathway enrichment analyses were performed to explore whether genes from various terms are significantly enriched than expected in the subgroups^25,26^.

### Ethics approval and consent to participate

The identities of patients are not provided in the TCGA and GEO databases, therefore, no approval and informed consent was required from the institutional review board.

## Results

### Differentially expressed glycolysis gene sets between tumor tissues and adjacent normal tissues

**Figure 1:**
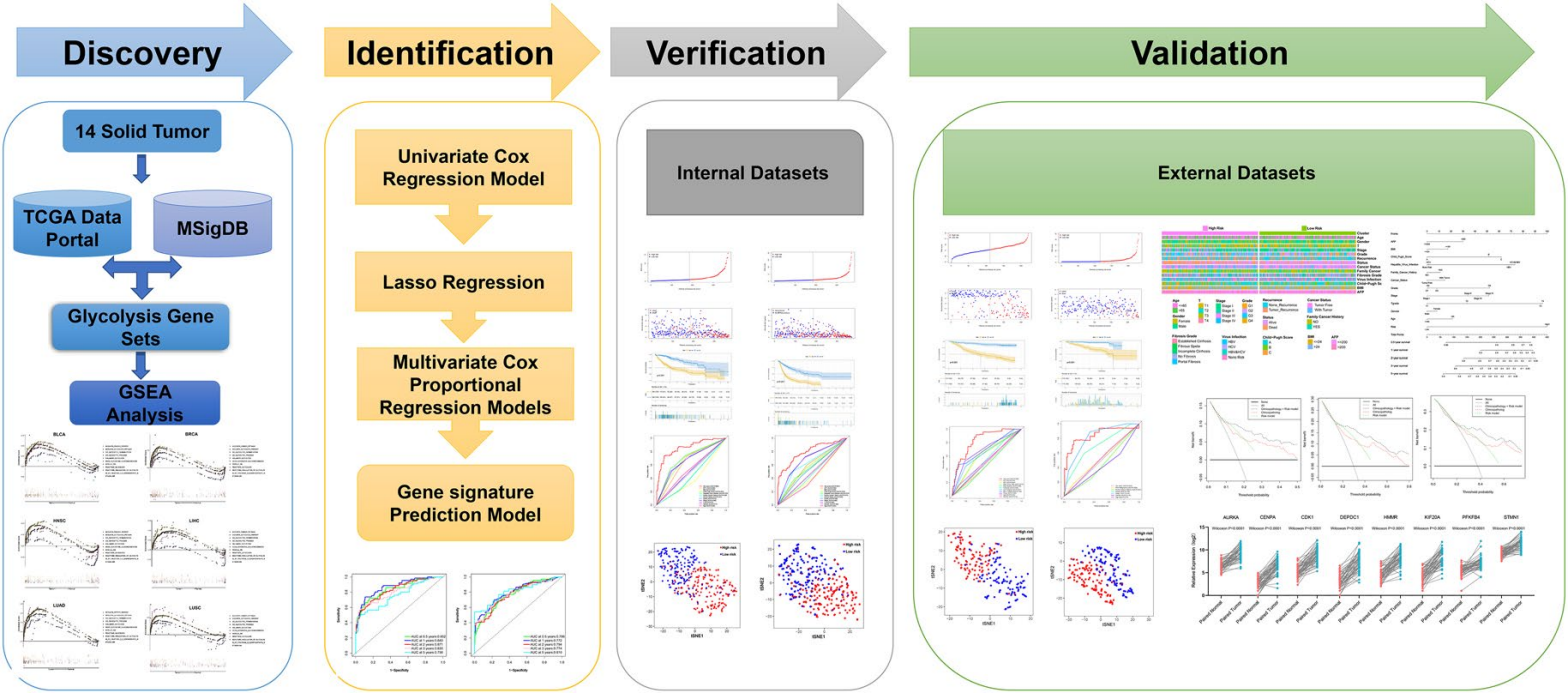
Panoramic flowchart for development and verification of glycolysis gene signatures.

**Figure 2:**
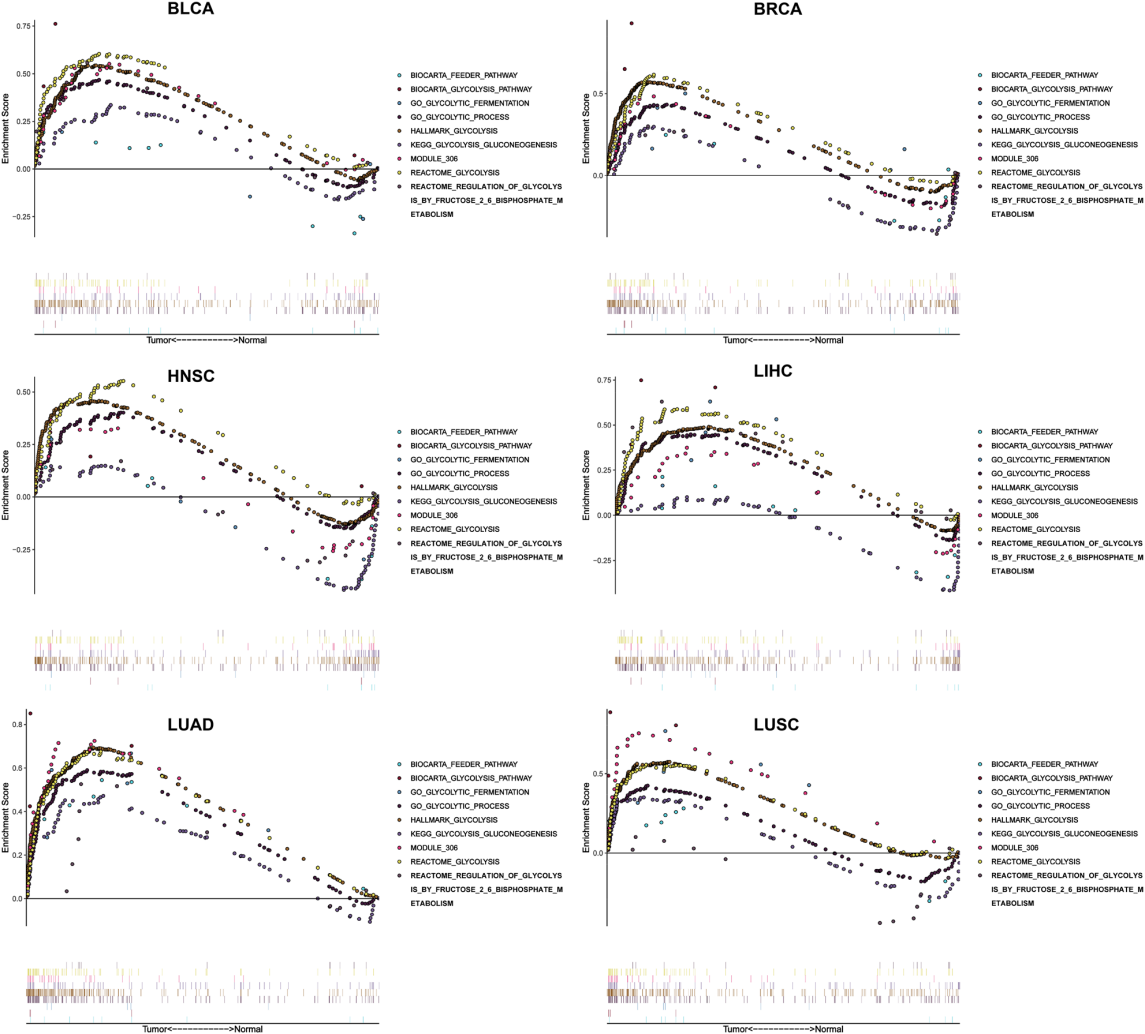
Enrichment curve of 9 glycolysis-related gene sets in 6 tumors (BLCA, BRCA, HNSC, LIHC, LUAD, LUSC), with FDR less than 0.05 as the statistical threshold.

The study process and analysis were presented as a panoramic flow chart (Fig. 1). A total of 12 solid tumors with complete clinical information and gene expression profiles including BLCA, BRCA, COAD, HNSC, KIRC, KIRP, LIHC, LUAD, LUSC, OV, PRAD, and THCA were included in the present study. All the above data were retrieved from TCGA and was subjected to normalization before performing GSEA. GSEA was performed on 9 gene sets associated with glycolysis process. GSEA was performed to explore whether these gene set variants were differentially expressed between tumors and their adjacent noncancerous tissues. One gene set with an FDR value less than 0.05 was selected for subsequent studies. At least one significant gene set was identified in BLCA, BRCA, HNSC, LIHC, LUAD, and LUSC (Fig. 2). The findings showed that FDR values of the 9 gene sets in COAD, KIRC, KIRP, OV, PRAD, and THCA were greater than 0.05. Distribution of the NES value and FDR q-value value of each gene set in the GSEA analysis is presented in Fig. S1a. Details on GSEA results are presented Fig. S2. A total of 6 solid tumors (BLCA, BRAC, HNSC, LIHC, LUAD, and LUSC) and corresponding core genes (CORE ENRICHMENT: YES) were used for further analysis (Table S1).

To further explore whether these core genes participated in the glycolysis process, GO and KEGG pathway analyses were performed using ClusterProfiler R package. The findings showed these genes are enriched in several pathways implicated in glucose metabolisms, such as pyruvate metabolic process, pyruvate biosynthetic process, a glycolytic process (Figure S1b–d), and glycolysis/gluconeogenesis (Figure S1e). These findings indicate that these core genes play important roles in glucose metabolism, mainly glycolysis.

### Construction and validation of a prognostic glycolysis associated-gene signature

**Figure 3:**
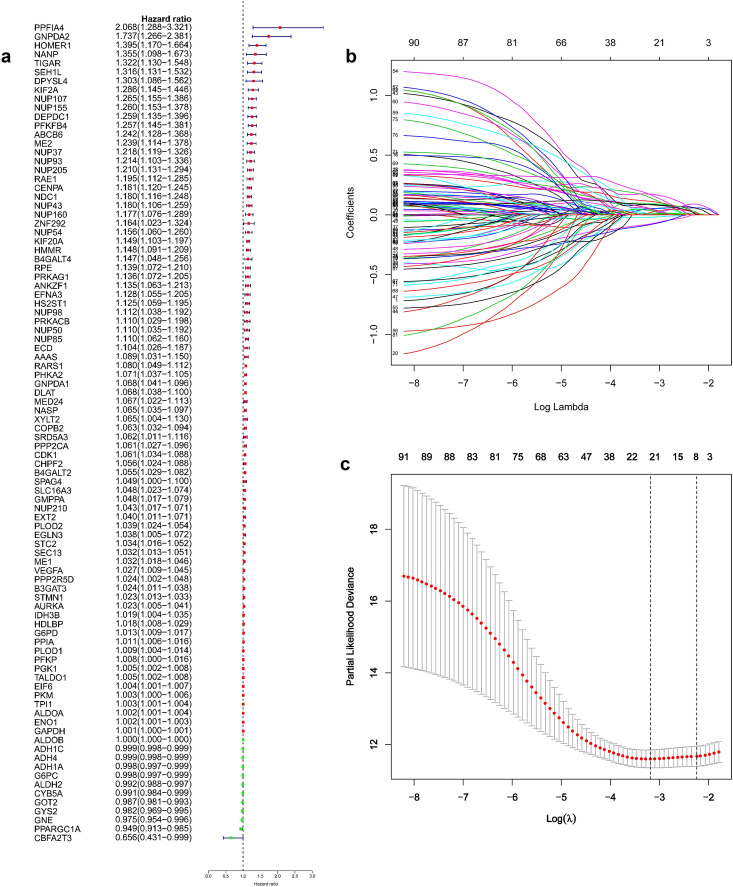
Identification of genetic signatures for HCC prognostic models. (**a**) Univariate cox regression analysis of 92 glycolytic genes correlated with OS in HCC patients. (**b**) LASSO coefficient profiles of the 92 OS-associated genes. (**c**) Adjustment parameter (λ) was selected through a 20-fold cross-validation procedure and plotted as a function of log (λ) in the LASSO model.

**Figure 4:**
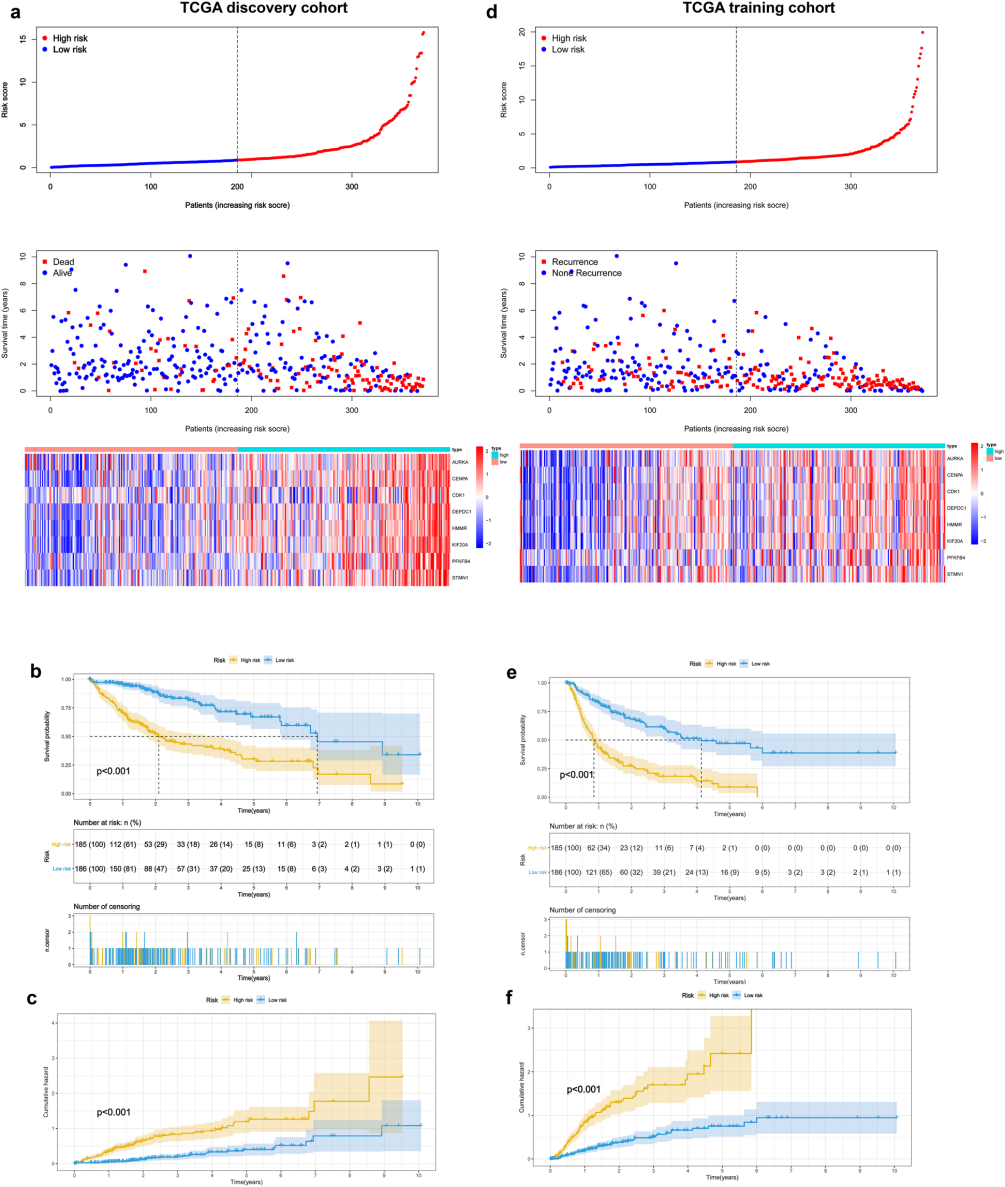
GRGPI signature serves as a promising risk prediction factor for overall survival (OS) and recurrence-free survival (RFS) in the TCGA cohort. (**a**,**b**) Distribution of risk score, survival status, and gene expression patterns of HCC patients in high- and low-risk groups for OS and RFS. (**c**,**d**) Kaplan–Meier plots for OS and RFS of the two risk groups in the TCGA cohort. (**e**,**f**) Performance of the cumulative event probability in the two risk subgroups.

**Figure 5:**
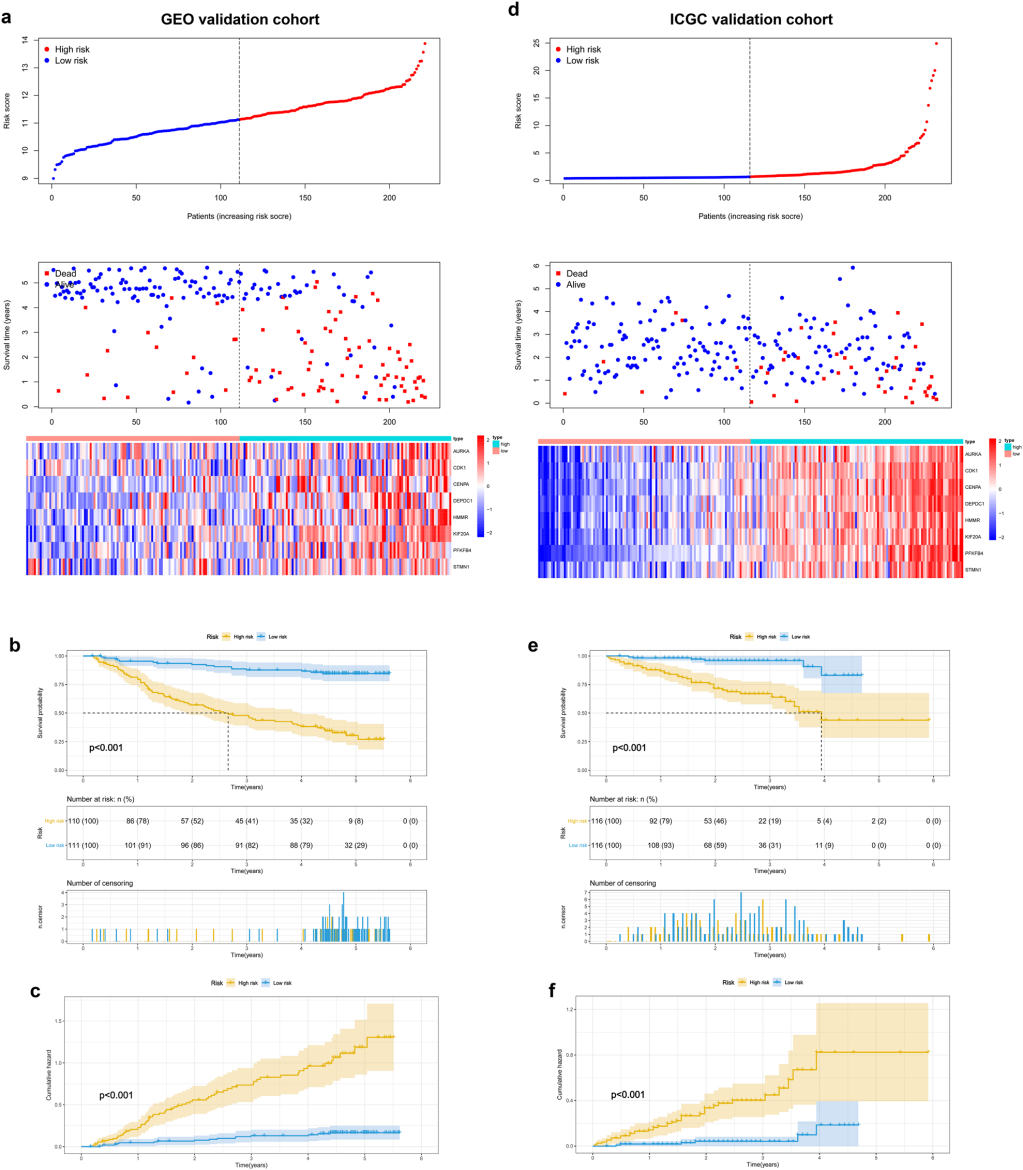
Performance of GRGPI signature in GEO and ICGC validation cohorts. (**a**,**b**) Distribution of risk score, survival status, and gene expression patterns of HCC patients in the 2 validation cohorts. (**c**,**d**) Kaplan–Meier plots for OS in the two risk subgroups. (**e**,**f**) Performance of the cumulative event probability in the two risk subgroups.

Relationship between core genes and OS was explored through univariate regression analysis and multivariate Cox-PH regression model with a stepwise procedure to identify important variables. The findings showed statistically significant gene signatures (GRGPI) were identified in BLCA, BRAC, HNSC, LIHC, LUAD, and LUSC (Table S2). These findings show that GRGPIs can be used to identify patients with adverse outcomes who would be classified as high-risk group based on these glycolysis gene-related classifiers. Further, the area under the time-dependent ROC curves (AUC) values were determined for each cancer type. The findings showed that LIHC had the highest AUC compared with that of BLCA, BRAC, HNSC, LUAD, and LUSC at 0.5- (0.852), 1- (0.840), 2- (0.871), 3- (0.830), and 5-year (0.756) (Fig. S3). A total of 92 glycolysis-related genes with significant correlations with overall survival were identified through univariate Cox regression analysis (Fig. 3a) in LIHC. Independent prognostic factors were restricted to variables that contributed significantly toward the final model coefficients based on the AIC and the model χ^2^ score to avoid overfitting and unnecessary complexity. Selected features were incorporated into a least absolute shrinkage and selection operator (LASSO) regression model to penalize for model complexity overfitting. A total of 8 genes (AURKA, DEPDC1, CDK1, CENPA, HMMR, KIF20A, PFKFB4, and STMN1) with nonzero LASSO coefficients (Fig. 3b,c). Multivariate analysis using Cox proportional hazard regression was used for virtual statistical weighting of the variables, and for determining their prognostic value. The risk score of 8 gene signatures was established as follows: Risk score = (0. 1224 × expression of AURKA) + (0.0534 × expression of CDK1) + (0.0920 × expression of CENPA) + (0.1323 × expression of DEPDC1) + (0.1140 × expression of HMMR) + (0.2425 × expression of KIF20A) + (0.1562 × expression of PFKFB4) + (0.0911 × expression of STMN1). Patients were grouped into high or low risk groups based on the median risk score of the TCGA discovery cohort. Distribution of risk scores, survival status, and gene expression profiles of patients varied significantly between the two subgroups (Fig. 4a). Kaplan–Meier survival analysis showed that survival of the low-risk group was significantly longer compared with that of the high-risk group (Fig. 4b, P < 0.001). Cumulative event probability curve showed that HCC patients in the high-risk group have a significantly higher probability of cumulative events during the entire follow-up period compared with that of low-risk patients (Fig. 4c, P < 0.001). We applied the classifier to assess whether the 8-mRNA signature can predict an individual or a specific HCC recurrence. TCGA dataset comprising recurrence events and recurrence time was used as an internal training cohort (TCGA training cohort). The prognostic evaluations of survival analysis for the 8-gene signature were based on TCGA recurrence-free survival (RFS) outcomes. Distribution of risk score, survival status, and gene expression patterns of patients are presented in Fig. 4d. The findings showed that patients with low-risk scores had longer RFS time compared with patients with high-risk scores (Fig. 4e, P < 0.001). Analysis of cumulative event occurrence curve showed a significant cumulative risk (HR) of HCC patients in the high-risk group compared with the low-risk group (Fig. 4f). The findings showed that the 8-gene signature can be used as a prognostic indicator for outcome and recurrence of HCC patients. Moreover, two independent analyses were conducted on the datasets from GEO and ICGC datasets. The findings showed that the 8-gene model effectively divided the two independent validation sets into two risk subgroups (Fig. 5a,d). Notably, the survival analysis and cumulative risk curve indicated that the high-risk group had a shorter OS and higher cumulative risk compared with the low-risk group (Fig. 5b,c and 5e,f). The findings showed robust prognostic value of the classifier in the 3 independent cohorts.

### Independent predictive value of the 8-mRNA signature

**Figure 6:**
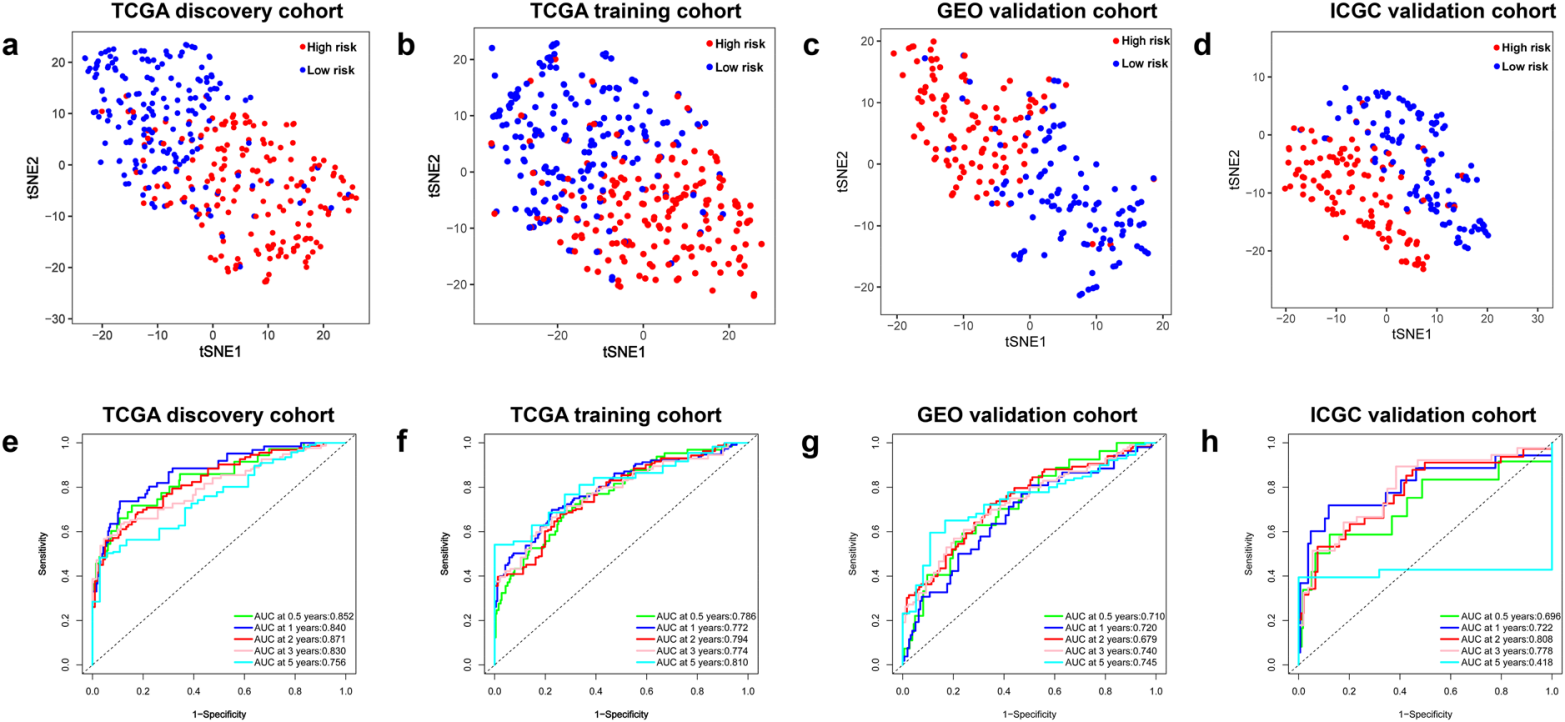
(**a**–**d**) T-SNE analysis showing the distribution of high-risk and low-risk patients in discrete directions in TCGA discovery cohort, TCGA training cohort, GEO validation cohort, and ICGC validation cohort. Time-dependent ROC curve analysis of the GRGPI model using the TCGA discovery cohort e, TCGA training cohort (**f**), GEO cohort (**g**), and ICGC cohort (**h**) for 0.5-, 1-, 2-, 3-, and 5- year OS.

Risk scores were calculated and used to develop predictive models for prediction of OS and RFS. To verify the assignments of sub-categories, t-SNE was performed to constraint the dimensionality of features. T-SEN analysis showed that the two risk subgroups were scattered in two discrete directions (Fig. 6a–d). Time‐dependent ROC curves were generated for TCGA discovery cohort, TCGA training cohort, GEO validation cohort, and ICGC validation cohort to estimate the prognostic accuracy of the 8 glycolysis-related signature in predicting 0.5-, 1-, 2-, 3- and 5-year OS and RFS. The findings showed high prognostic accuracy of the 8-signature model (Fig. 6e–h).

**Figure 7:**
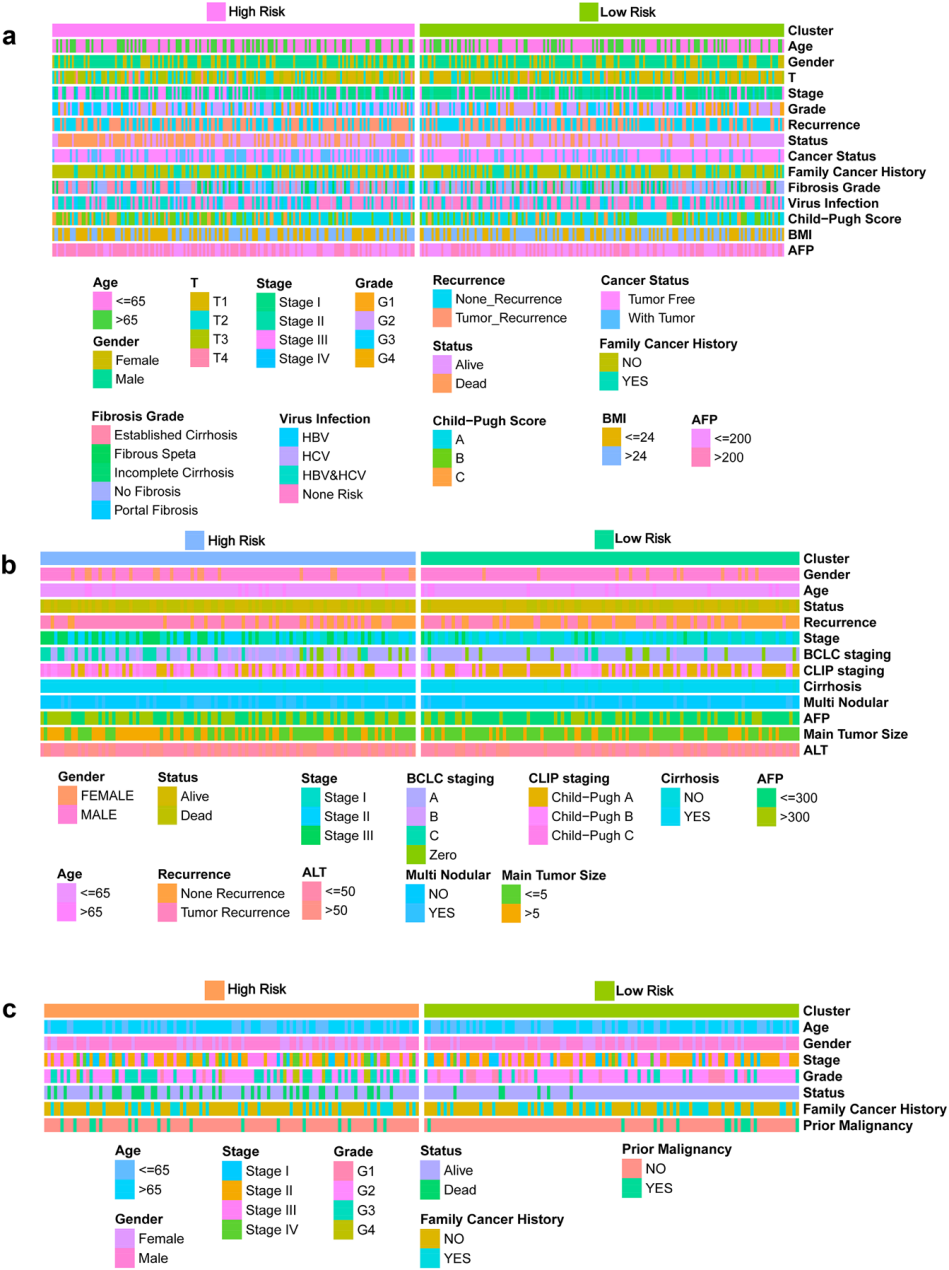
Clinical characteristics of HCC subclasses in the (**a**) TCGA, (**b**) GEO, and (**c**) ICGC cohorts.

**Figure 8:**
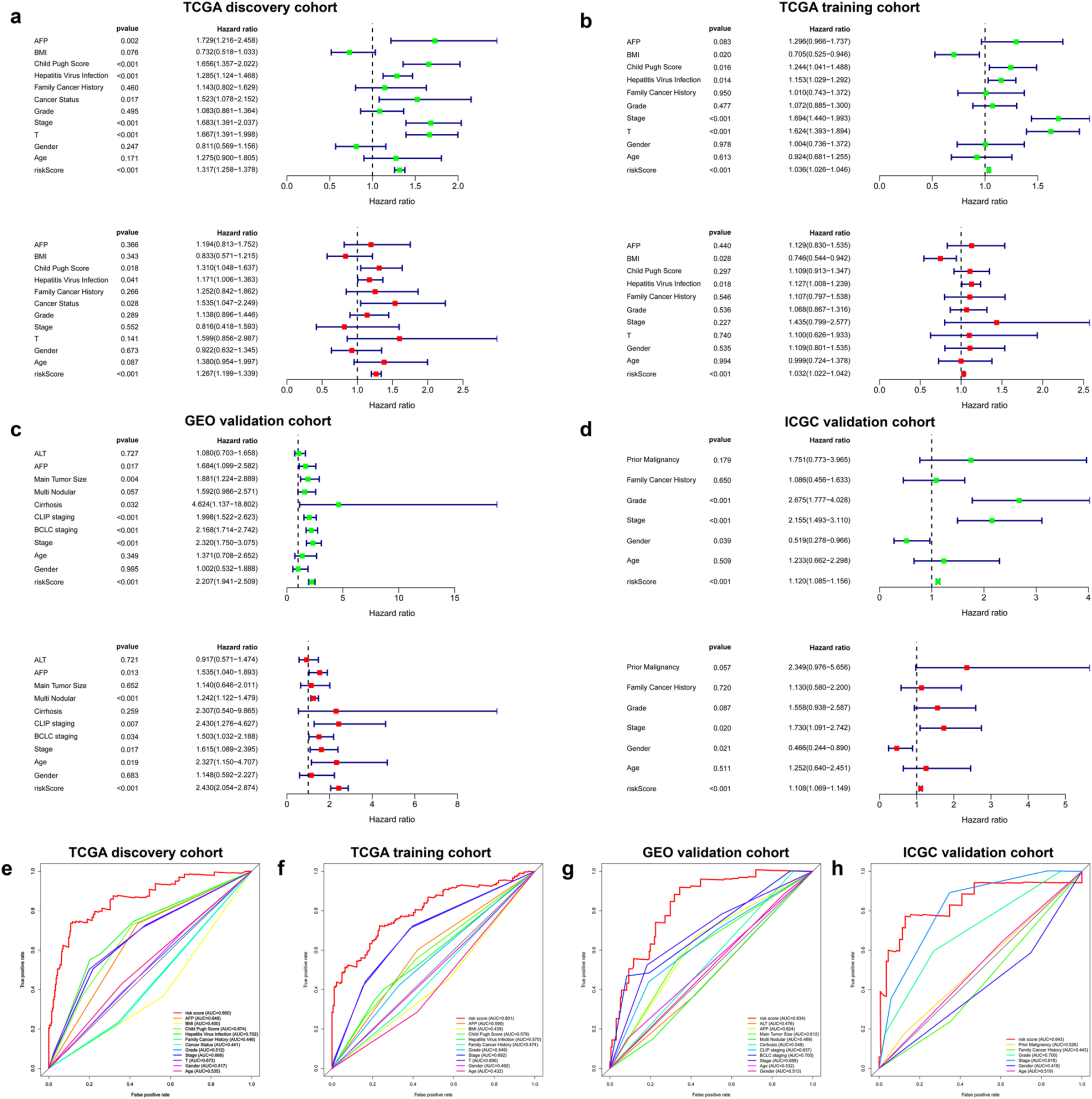
(**a**–**d**) Univariate and multivariate Cox regression analysis based on all variables for prediction of OS and RFS in the 4 cohorts. Green dots represent the HR value of univariate cox analysis, and red dots represent the HR value of multivariate cox analysis. Blue lines indicate the standard error (SE) of HR. Multiple ROC curves comparing the predictive power of the GRGPI model and other clinicopathological features based on TCGA discovery cohort (**e**), TCGA training cohort (**f**), GEO cohort (**g**), and ICGC cohort (**h**).

The explanation for many clinical situations one can identify some standard variables that have previously been demonstrated to have prognostic value and are generally measured for most patients having the particular diagnosis. Further, tumor‐related clinicopathological variables associated with the classifier in the current study was explored based on TCGA (Fig. 7a, Table S3), GEO (Fig. 7b, Table S4), and ICGC (Fig. 7c, Table S5) cohorts. Patient clinicopathologic characteristics are presented in Table 1. Pearson's correlation analysis showed several significant correlations between clinicopathological characteristics and HCC risk subtypes in the three independent cohorts. The findings showed that T classification (P = 0.0032), stage (P < 0.001), grade (P = 0.0175), family cancer history (P = 0.0359), AFP level (P = 0.0147), cancer status (P < 0.001), recurrence event (P < 0.001) and patient status were significantly correlated with HCC risk groups in the TCGA discovery cohort (P < 0.001). In addition, there was a significant correlation was observed between T classification (P = 0.0095) and Stage (P = 0.0033) between HCC subgroups in TCGA training set (Table S3). Similarly, analysis of the GEO and ICGC cohorts showed that some important clinicopathological characteristics had significant correlations with HCC subgroups. More detailed results are shown in Table S4 and Table S5. To verify the independence of GRGPI, a Cox proportional hazard regression analysis was performed using the TCGA, GEO, and ICGC cohorts (Table 2). The adjustment results of clinical variables showed that risk score remained an independent prognostic factor, indicating its robust predictive ability for OS (HR = 1.267, P < 0.001) and RFS (HR = 1.027, P < 0.001) of HCC patients (Fig. 8a,b). Findings of the Cox regression model showed that, some clinical-pathological factors (cancer status (P = 0.017), hepatitis virus infection (P = 0.041), and Child–Pugh score (P = 0.011) for OS; cancer status (P < 0.001), hepatitis virus infection (P = 0.017), and BMI (P = 0.009) for RFS) were independent poor prognostic factors. These variables were thus valuable for risk stratification in pathological subgroups as shown in Fig. 8a,b. In addition, KM survival analysis revealed that disease-specific survival rates were significantly different in some pathological subgroups, such as T classification (T1–2 vs T3-4, P < 0.001), stage (Stage I–II vs III–IV, P < 0.001), cancer status (Tumor Free vs With Tumor, P = 0.011), hepatitis virus infection (HVI Negative vs Positive, P = 0.008), Child–Pugh Score (A/B vs C, P < 0.001) and AFP (≤ 200 vs > 200, P = 0.002) for TCGA OS (Figure S4). These findings were consistent with the findings from univariate Cox regression for OS with adjustments for prognostic factors (Fig. 8a, Table S3). In addition, the index independently predicted the OS of GEO (HR (95% CI) = 2.430 (2.054–2.874), P < 0.001) (Fig. 8c) and ICGC cohorts (HR (95% CI) = 1.108 (1.069–1.149), P < 0.001) (Fig. 8d). These findings indicate GRGPI is an independent prognostic factor for HCC patients.

**Table 1:** Clinicopathological characteristics of HCC patients included in the TCGA, GEO, and ICGC cohorts.

Variables	TCGA Cohort (n = 371)	ICGC Cohort (n = 232)	GEO Cohort (n = 221)
**Age**			
≤ 65	233	90	200
> 65	138	142	21
**Gender**			
Female	121	61	30
Male	250	171	191
**T classification**			
T1	183	N/A	N/A
T2	95	N/A	N/A
T3	80	N/A	N/A
T4	13	N/A	N/A
**Stage classification**			
Stage I	179	36	93
Stage II	93	106	78
Stage III	92	71	50
Stage IV	7	19	0
**Grade**			
G1	57	22	N/A
G2	178	142	N/A
G3	124	59	N/A
G4	12	9	N/A
**BCLC stage**			
Zero	N/A	N/A	21
A	N/A	N/A	149
B	N/A	N/A	22
C	N/A	N/A	29
**Status**			
Alive	241	189	131
Dead	130	43	90
**Recurrence**			
No	191	N/A	100
Yes	180	N/A	121
**Cancer status**			
Tumor free	250	N/A	N/A
With tumor	121	N/A	N/A
**Family cancer history**			
No	251	152	N/A
Yes	120	80	N/A
**Prior malignancy**			
No	N/A	202	N/A
Yes	N/A	30	N/A
**Multi nodular**			
No	N/A	N/A	176
Yes	N/A	N/A	45
**Cirrhosis**			
No	N/A	N/A	18
Yes	N/A	N/A	203
**Fibrosis grade**			
No fibrosis	127	N/A	N/A
Incomplete cirrhosis	15	N/A	N/A
Established cirrhosis	111	N/A	N/A
Fibrous speta	42	N/A	N/A
Portal fibrosis	76	N/A	N/A
**Hepatitis virus infection**			
None risk	195	N/A	0
HBV	61	N/A	221
HCV	18	N/A	0
HCV and HBV	97	N/A	0
**Child**–**Pugh Score**			
A	198	N/A	97
B	99	N/A	75
C	74	N/A	49
**BMI**			
≤ 24	174	N/A	N/A
> 24	197	N/A	N/A
**AFP level**			
Low	190	N/A	121
High	181	N/A	100
**ALT level**			
≤ 50	N/A	N/A	130
> 50	N/A	N/A	91
**Tumor size**			
≤ 5	N/A	N/A	140
> 5	N/A	N/A	81

*BCLC stage* Barcelona Clinic Liver Cancer Stage, *AFP* alpha fetoprotein, *ALT* alanine aminotransferase.

**Table 2:** Univariable and multivariable analyses for each clinical variables in TCGA, GEO, and ICGC cohort.

TCGA		Patients (n)	Univariate analysis			Multivariate analysis		
Variables			HR	95% CI	P value	HR	95% CI	P value
AFP	≤ 200/> 200	190/181	1.73	1.22–2.46	2.30E−03	1.19	0.81–1.75	3.66E−01
BMI	≤ 24/> 24	174/197	0.73	0.52–1.03	7.61E−02	0.83	0.57–1.22	3.43E−01
Child Pugh Score	A/B/C	198/99/74	1.66	1.36–2.02	7.10E−07	1.31	1.05–1.64	1.76E−02
Hepatitis virus infection	None risk/HBV/HCV/HCV and HBV	195/61/18/97	1.28	1.12–1.47	2.34E−04	1.17	1.01–1.36	4.15E−02
Family cancer history	No/yes	251/120	1.14	0.80–1.63	4.60E−01	1.25	0.84–1.86	2.66E−01
Cancer status	Tumor free/with tumor	250/121	1.52	1.08–2.15	1.71E−02	1.53	1.05–2.25	2.81E−02
Grade	G1/G2/G3/G4	57/178/124/12	1.08	0.86–1.36	4.95E−01	1.14	0.90–1.45	2.89E−01
Stage	Stage I/II/III/IV	179/93/92/7	1.68	1.39–2.04	8.92E−08	0.82	0.42–1.59	5.52E−01
T	T1/T2/T3/T4	183/95/80/13	1.67	1.39–2.00	3.16E−08	1.60	0.86–2.99	1.41E−01
Gender	Female/male	121/250	0.817	0.60–1.16	2.47E−01	0.92	0.63–1.35	6.73E−01
Age	≤ 65/> 65	233/138	1.277	0.90–1.80	1.71E−01	1.38	0.95–2.00	8.73E−02
Risk score	High/low	185/186	1.32	1.26–1.38	1.18E−32	1.27	1.20–1.34	4.08E−17

GEO		Patients (n)	Univariate analysis			Multivariate analysis		
Variables			HR	95% CI	P value	HR	95% CI	P value
AFP	≤ 200/> 200	190/181	1.30	0.97–1.74	8.34E−02	1.32	0.96–1.81	8.40E−02
BMI	≤ 24/> 24	174/197	0.71	0.53–0.95	1.98E−02	0.66	0.48–0.90	1.98E−02
Child Pugh Score	A/B/C	198/99/74	1.66	1.04–1.50	1.65E−02	0.99	0.82–1.20	1.65E−02
Hepatitis virus infection	None risk/HBV/HCV/HCV and HBV	195/61/18/97	1.16	1.03–1.30	1.40E−02	1.16	1.03–1.31	1.40E−02
Family cancer history	No/yes	251/120	1.01	0.74–1.37	9.50E−01	0.97	0.69–1.34	9.50E−01
Cancer status	Tumor free/with tumor	250/121	3.96	2.93–5.35	3.61E−19	4.12	2.99–5.66	3.61E−19
Grade	G1/G2/G3/G4	57/178/124/12	1.07	0.89–1.30	4.77E−01	1.02	0.83–1.26	4.77E−01
Stage	Stage I/II/III/IV	179/93/92/7	1.69	1.44–1.99	2.13E−10	1.35	0.81–2.25	2.13E−10
T	T1/T2/T3/T4	183/95/80/13	1.62	1.39–1.89	5.97E−10	1.12	0.69–1.83	5.97E−10
Gender	Female/male	121/250	1.01	0.74–1.37	9.78E−01	1.23	0.89–1.71	9.78E−01
Age	≤ 65/> 65	233/138	0.92	0.68–1.26	6.13E−01	0.85	0.61–1.18	6.13E−01
Risk score	High/low	185/186	1.04	1.03–1.05	4.32E−13	1.03	1.02–1.4	4.32E−13

ICGC		Patients (n)	Univariate analysis			Multivariate analysis		
Variables			HR	95% CI	P value	HR	95% CI	P value
ALT	≤ 50/> 50	130/91	1.08	0.70–1.66	7.27E−01	0.92	0.57–1.47	7.21E−01
AFP	≤ 300/> 300	121/100	1.68	1.10–2.58	1.67E−02	1.56	1.04–1.89	1.26E−02
Main tumor size	≤ 5/> 5	140/81	1.88	1.22–2.89	3.93E−03	1.14	0.65–2.01	6.52E−01
Multi nodular	No/yes	176/45	1.59	0.99–2.57	5.72E−02	1.24	1.12–1.48	4.57E−05
Cirrhosis	No/yes	18/203	4.62	1.14–18.8	3.24E−02	2.31	0.54–9.87	2.59E−01
CLIP staging	Child–Pugh A/B/C	97/75/49	1.20	1.52–2.62	6.04E−07	2.43	1.28–4.63	6.90E−03
BCLC staging	Zero/A/B/C	21/149/22/29	2.17	1.71–2.74	1.09E−10	1.50	1.03–2.19	3.35E−02
Stage	Stage I/II/III/IV	93/78/50/0	2.32	1.75–3.08	4.79E−09	1.62	1.09–2.40	1.72E−02
Gender	Female/male	30/191	1.02	0.53–1.89	9.95E−01	1.15	0.59–2.28	6.83E−01
Age	≤ 65/> 65	200/21	1.37	0.71–2.65	3.49E−01	2.33	1.15–4.71	1.88E−02
Risk score	High/low	110/111	2.21	1.94–2.51	1.31E−33	2.43	2.05–2.87	4.08E−25

Further stratified analysis was performed to explore independence of the model within the same subgroups of clinicopathological features. Taking advantage of the clinicopathological parameters, TCGA discovery cohort was divided into subgroups based on clinical-pathological features, such as gender (Male/Female), age (≤ 65/ > 65), grade (G1–2/G3–4), stage (Stage I–II/III–IV), T classification (T1–2/3–4), tumor status (Tumor Free/With Tumor). After stratification, the 8-mRNA signature accurately divided cohort into low- and high-risk patients (Fig. S5 and Fig. S6). Similar were obtained from the GEO (Fig. S7–S9) and ICGC (Fig. S10, S11) cohorts.

A multiple ROC curve analysis was performed to determine the sensitivity and specificity of the OS/RFS prognostic model and other clinical pathology variables. The 8-mRNA model was used for analysis of the TCGA discovery cohort, TCGA training cohort, GEO, and ICGC cohort, and the prediction quality was compared by evaluating the area under the ROC curve to determine its performance. Multivariate Cox regression and AUC analyses showed that the prognostic model was an independent prognostic indicator with high accuracy (TCGA discovery cohort: AUC = 0.860; TCGA training cohort: AUC = 0.801; GEO validation cohort: AUC = 0.834; ICGC validation cohort: AUC = 0.843; Fig. 8e–h). The findings showed that the risk score model performed better compared with other clinical pathology variables for prognostic prediction of HCC patients. These findings indicated that GRGPI signatures have a predominately higher favorable value compared with other parameters in predicting OS and RFS of HCC patients.

### Landscape of immune infiltration in HCC risk subgroups

**Figure 9:**
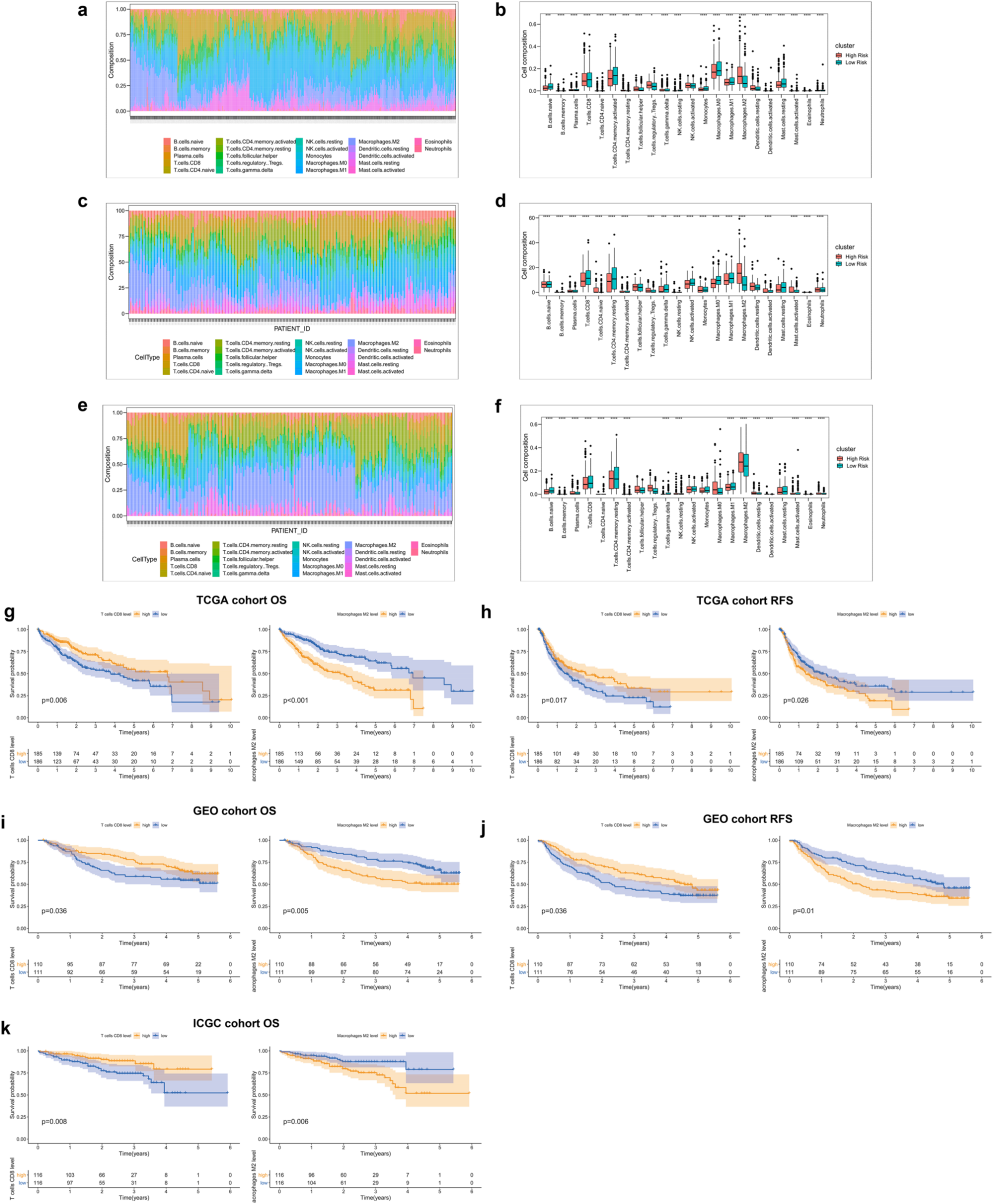
Global inflammatory landscape in high and low-risk groups of HCC patients in TCGA (**a**), GEO (**c**), and ICGC (**e**) cohorts. (**b**) Boxplots showing panoramic distribution of immune cells between the 2 risk subgroups (B, TCGA; D, GEO; F, ICGC), and significance was determined by the Wilcoxon test. (**d**) KM-plot showing imbalance of 2 immune cells associated with the OS and RFS status of HCC patients in the 3 cohorts, patients were divided based on the median of CD8+ T cells or Macrophage M2.

Immune infiltration was explored to characterize their immunological characteristics owing to the significant differences between subtypes. The CIBERSORT algorithm was used to determine the abundance of 22 immune-related cell types and the findings were presented as heatmaps and box plots for TCGA (Fig. 9a,b), GEO (Fig. 9c,d), and ICGC (Fig. 9e,f) cohorts, respectively. Notably,, frequency of CD8+ T cells in the low-risk group was significantly higher compared with that in the high-risk group, whereas proportion of M2 macrophages was higher in the high-risk group compared with that of the low-risk group, in 3 independent cohorts. Analysis of tumor immune infiltration levels of each patient showed that high CD8+ T cell levels was correlated with better survival, whereas high levels of M2 cells indicated worse OS and RFS in HCC tissues (Fig. 9g–k).

### WGCNA and GSEA analysis

**Figure 10:**
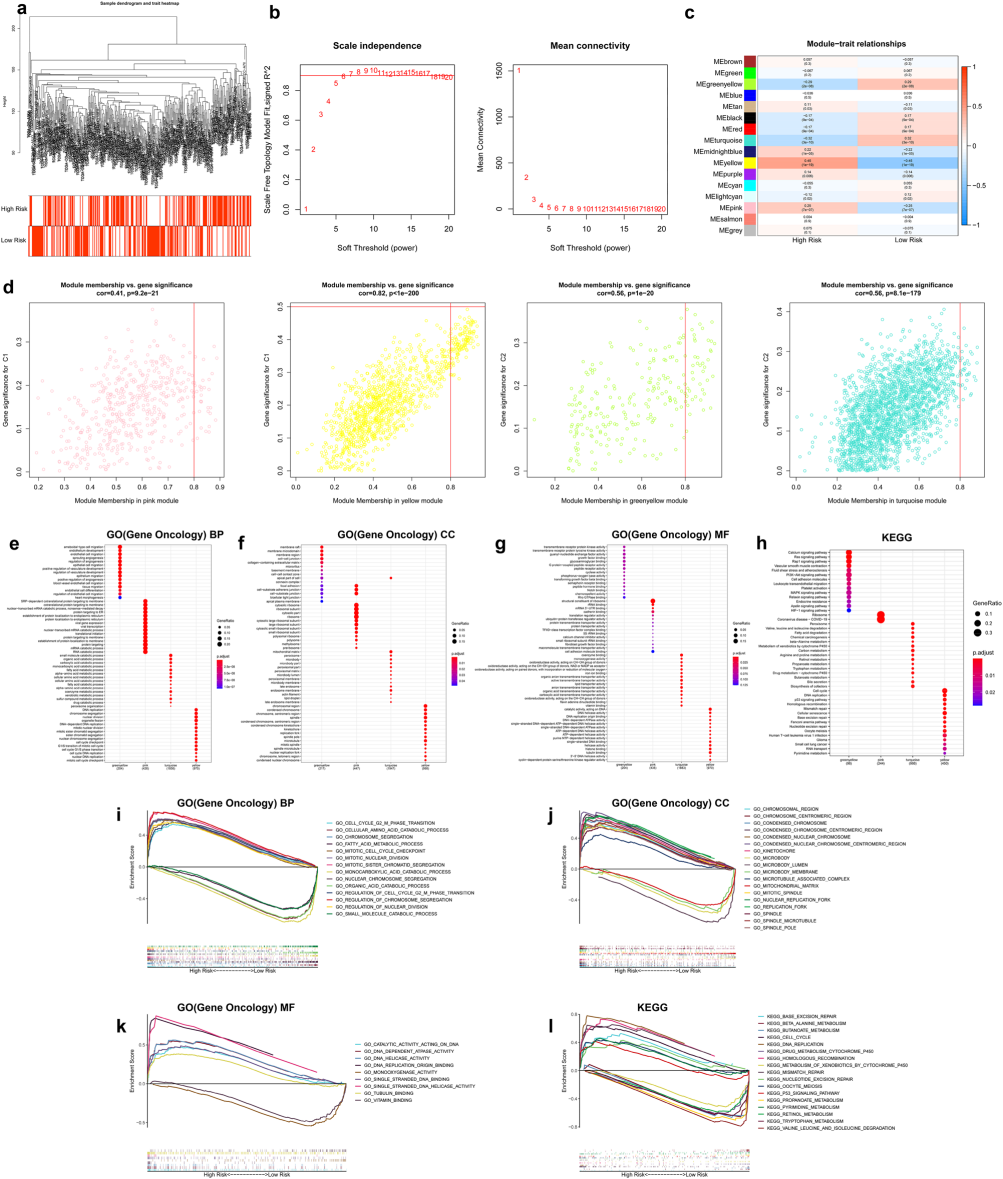
Identification of subtype-specific gene profile and biological function by WGCNA and GSEA in TCGA cohort. (**a**) Clustering dendrograms of HCC samples. (**b**) Analysis of the scale-free fit index (left) and average connectivity (right) under various soft threshold powers (β = 7). (**c**) Correlations between the eigenvector values of 16 modules and the subtype characteristics. (**d**) Scatter diagram of eigengenes in the top2 modules for each subtype. Heatmap showing the top 15 terms significantly enriched (**e**) GO(BP), (**f**) GO(CC), (**g**) GO(MF), and (**h**) KEGG for each WGCNA module. (**i**–**l**) The multiple GSEA curves describe some important biological terms, which are consistent with the results of WGCNA analysis.

WGCNA and GSEA analysis were performed to identify differential gene expression patterns between different subgroups. Notably, no outlier samples were detected based on average clustering (Fig. 10a). The soft threshold β was set at 7 to determine a scale-free network (Fig. 10b). Genes were assigned to 16 modules, and gray modules included genes that could not be clustered (Fig. 10c). Two gene modules highly correlated with high- (pink, yellow) and low-risk (greenyellow, turquoise) groups were identified (Fig. 10d). Further, GO and KEGG analyses were performed to identify the potential biological significance of related TOP2 modules in different subgroups (Fig. 10e–h). Moreover, GSEA analysis was performed based on the overall TCGA-LIHC expression profiles. The terms identified in both WGCNA and GSEA analysis results are presented in Fig. 10i–l. Terms related to cell cycle transition, chromatin separation, DNA replication, and DNA helicase activity were significantly enriched in the high-risk group.

### Further verification of the 8-gene signature

**Figure 11:**
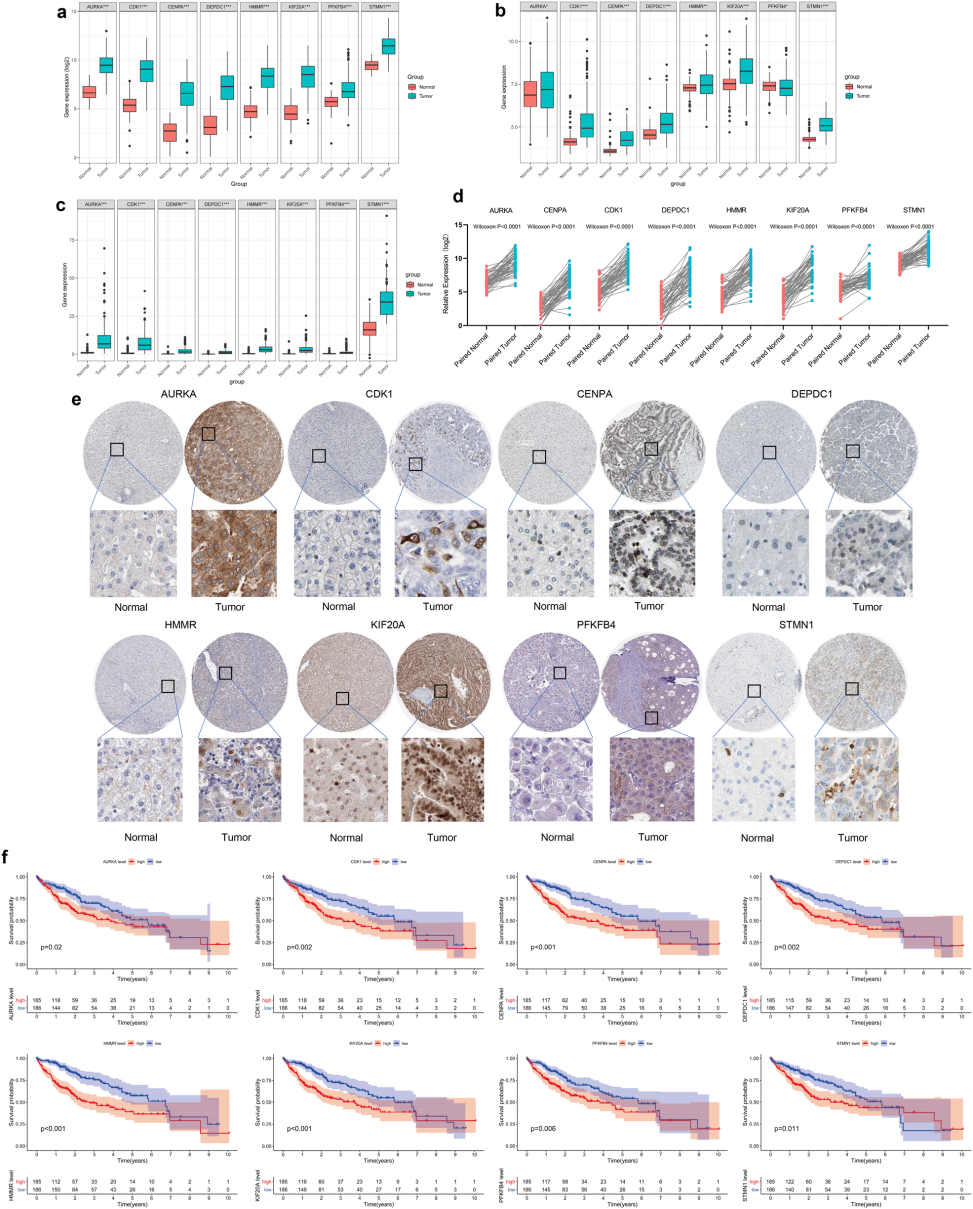
Imbalance of prognostic signature at the gene level and protein level. Expression of three 8-gene signatures in normal and tumor tissues at the transcriptome level in TCGA (**a**), GEO (**b**), and ICGC (**c**) cohorts. (**d**) Comparing the expression of 8 gene signature in 50 cancer and paired paracancerous. (**e**) Staining intensity of the 8-gene signature in HCC pathological tissue and corresponding normal liver tissue. (**f**) The relationship between the expression of 8 central genes and the survival time of patients with HCC.

Additional verification was performed at the transcriptome level and protein level to further explore the reliability of the 8-gene signatures. Expression of the 8 gene was explored based on the TCGA, GEO, and ICGC databases. The findings showed a general trend that these 8 genes included in the signature were upregulated in HCC tumor tissues (Fig. 11a–c). Results from paired t-test (50 tumor tissues and paired normal adjacent tissues ) exhibited consistent trend (Wilcoxon test P value < 0.0001) (Fig. 11d). Furthermore, expression levels of the 8 genes were determined at the protein level using immunohistochemistry (IHC), based on the Human Protein Atlas database. IHC showed upregulation of the eight proteins in HCC tissues. Moderate or high staining intensity of the 8 proteins in HCC tissues contrasted sharply with the low intensity or lack of staining in normal tissues (Fig. 11e). In addition, our research found that there were significant impact on the relationship between the expression of 8 central genes and the survival time of patients with HCC (Fig. 11f).

### Development and verification of a personalized nomogram

**Figure 12:**
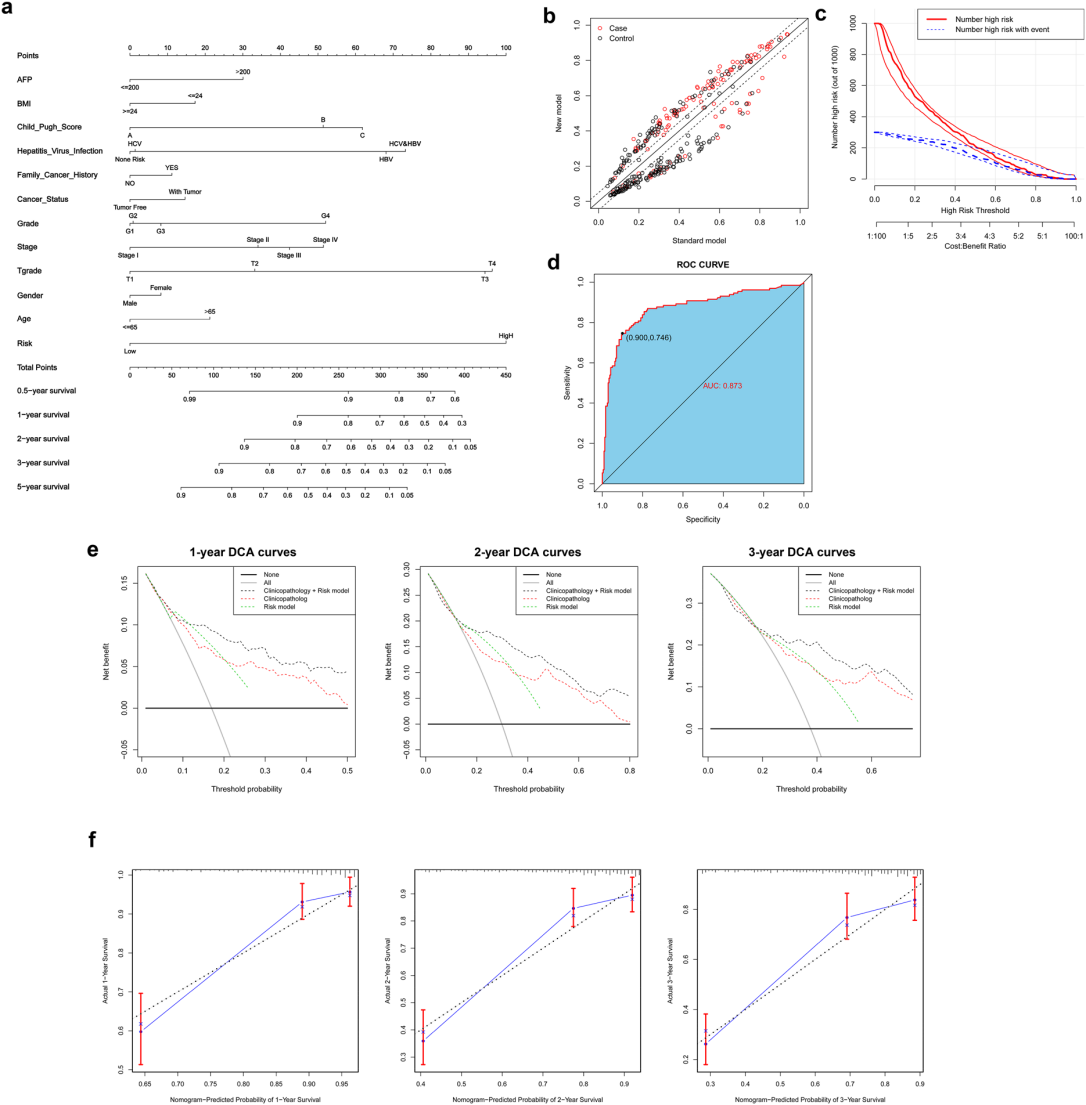
Performance of a personalized nomogram based on GRGPI and clinicopathological features. A nomograph for predicting overall survival probability of HCC patients. (**b**,**c**) The nomogram model improves identification of high-risk patients, and 371 HCC patients are reclassified between the standard model and the Nomogram model. (**d**) ROC curve for the nomogram model. (**e**) Decision curve analyses of the nomogram for 1-, 2-, and 3-year OS. (**f**) Calibration curves of 1‐, 2-, and 3‐year OS for HCC patients in the TCGA discovery cohort.

A nomogram integrating GRGPI and clinicopathological characteristics was constructed using TCGA, GEO, and ICGC cohorts to provide clinicians with a portable quantitative table for predicting prognosis of liver cancer patients. The risk score contributed the largest risk point in the TCGA cohort, compared with other clinicopathological characteristics, followed by T classification, hepatitis virus infection, Child–Pugh score and stage, etc. (Fig. 12a). A total of 371 patients were reclassified in the new nomogram model for OS NRI (net reclassification index) = 0.415 (Fig. 12b,c). ROC analysis showed that the nomogram had high accuracy, and was a good predictor of patient survival, with an AUC value of 0.873 (Fig. 12d). Decision curve analysis showed that the novel nomogram had more net benefit across the range of decision threshold probabilities compared with the Risk score model and integrated clinicopathology model (Fig. 12e). Calibration curves showed a stable agreement between the prediction by the nomogram and the actual observation for 1-, 2-, and 3-year OS (Fig. 12f). In addition, the novel nomogram model integrated GRGPI and clinicopathological features and showed good agreement between the predicted and observed survival probabilities in the GEO (AUC = 0.854) and ICGC (AUC = 0.863) cohorts (Fig. S12 and Fig. S13).

## Discussion

Hepatocellular carcinoma (HCC) is a highly malignant tumor that accounts for approximately 90% of the total primary liver cancer cases^25,26^. It is the most common malignancy and the leading cause of cancer mortality globally^27^. Studies report a high incidence of HCC in China. The World Cancer Report released by the World Health Organization in 2019 indicated that new cases of liver cancer in China account for half of the global new cases, and the total number of death accounts for more than half of the global deaths cases^28^. Therefore, several studies have explored treatment approaches for hepatocellular carcinoma. Surgical treatment is the conventional treatment method HCC^29–31^. However, surgery is not suitable for treatment of some patients owing to tumor anatomical location, tumor size, tumor number, insufficient liver residual volume, or extrahepatic metastasis^32^. Nonsurgical therapy approaches are currently available for treatment of liver cancer patients. Recent development of medical technology and equipment has significantly improved management strategy for HCC^33^. These approaches enable regulation of various ontogenetic modifications including inflammation, immune suppression, and direct modulation of host cell behavior. Cancer cells undergo adaptive metabolic programming to maintain their distinctive metabolic state of continuous proliferation. Metabolic rewiring in cancer cells makes them highly dependent on specific metabolic enzymes or processes, thus it is a potential target for designing cancer-specific therapeutics^34,35^. Glycogen metabolism is an important metabolic process in the liver. Reprogramming of glucose metabolism significantly promotes aberrant proliferation and survival in HCC cells compared with non-cancerous cells^36–38^. Several enzymes and proteins involved in the process of HCC can undergo structural, functional, and expression changes to achieve metabolic reprogramming, which in turn controls the entire glycogen metabolism network thus promoting HCC growth^39–42^. The current study explored dysregulation of expression patterns of glycolysis-related genes to determine the metabolic activity of tumors in hypoxic mode. Comprehensive bioinformatics analyses were conducted using gene sets containing genes that encode key glycolytic enzymes. Core genes from GSEA that were significantly enriched in tumor tissues were selected for subsequent analysis. Transcriptomic analyses were conducted followed by K–M analysis to evaluate the correlation between expression of glycolysis-associated gene signatures and patient prognosis in 12 solid tumors. A total of 8 independent prognostic genes in HCC, including AURKA, CDK1, CENPA, DEPDC1, HMMR, KIF20A, PFKFB4, and STMN1 were identified through multivariate Cox-PH regression analysis. A prognostic model was developed based on the 8-gene signature and the performance was verified using 3 independent verification cohorts. The findings showed that gene signatures implicated in glycolysis pathway can accurately predict the poor prognosis and recurrence of HCC patients. Notably, the prognostic model showed more accurate predictive ability and was superior compared with other pathological features. To explore clinical application of the GRGPI-based risk model, a nomogram integrating multiple important clinicopathological characteristics was established, which can be used as a powerful and easy-to-use tool for evaluating survival probability of HCC patients.

Several studies report that some gene signatures are derived from glucose metabolism, including several genes that are critical for glycolysis and are overexpressed in glycolytic cancer cells. Aurora kinase A (AURKA) is an important regulatory protein involved in regulation of chromosome congression/alignment, regulation of chromosome segregation, and regulation of spindle dynamics^43,44^. In addition to the effects in the cancer environment, AURKA actively promotes DNA repair and acts as a transcription factor to promote cell migration and invasion^44,45^. It is located in the mitochondrial membrane where it regulates mitochondrial dynamics and ATP production^46,47^. AURKA is an effective prognostic indicator that probably integrates multiple oncogenic events in progression of tumors^43,48,49^. CDK1 (Cyclin-dependent kinase 1) is a serine/threonine-like protein kinase that plays an essential role in controlling cell proliferation at the G2/M point of the cell cycle. Studies report that high CDK1 expression level is an independent predictor for tumor recurrence in one and five years, and compounds that target CDK1 can be novel antitumor reagents^50–52^. CENPA is overexpressed in several cancers, and plays an auxiliary but important role in cancer pathogenesis, progression, distant metastases and invasion angiogenesis, etc^53,54^. Previous studies report that CENPA is significantly overexpressed in hepatocellular carcinoma (HCC) tumor tissue. CENPA is associated with overall survival (OS), disease-free survival (DFS), relapse-free survival (RFS), and progression-free survival (PFS) of HCC^55–57^. Key functions and potential regulatory pathways of DEP domain-containing protein 1 (DEPDC1), a newly discovered gene related to cancer and cell cycle, have been reported in bladder cancer, and other human cancers, such as breast cancer and prostate cancer. Previous studies report overexpression of DEPDC1 in several tumors and reported that it drives tumor pathogenesis through multiple potential mechanisms^58–60^. HMMR and KIF20A are important regulators of mitosis and exhibit oncogenic properties in various cancers through multiple mechanisms^61–64^. The Warburg pathway enzyme 6-phosphofructo-2-kinase/fructose-2,6-bisphosphatase 4 (PFKFB4) is implicated in regulation of diverse biological processes and plays an important role in regulating glucose metabolism and guiding macromolecule biosynthesis to promote proliferation of cancer cells. Several studies screened and identified PFKFB4 as a poor prognostic factor for multiple tumors through high-throughput analysis^65–67^. STMN1 is an oncogene and its aberrant upregulation is closely related to different kinds of tumors^68,69^. STMN1 is an independent predictive factor of poor outcome, it is upregulated in hepatocellular carcinoma and promotes migration, invasion, and EMT by activating PI3K/AKT pathway^69^.

Dysregulation of glycolytic pathway is a hallmark of oncogenic potential in tumor biology. Reprogrammed glycolytic and mitochondrial pathways are hallmarks of altered energy generation system of malignant cells, and lead to abnormal survival and proliferation of tumor cells^70^. Metabolic mode switching from aerobic oxidation to anaerobic glycolysis is an important characteristic of hepatocellular carcinoma. The Warburg effect results in accumulation of the lactic acid, as the final product of glycolysis. The acidic microenvironment thus mediates immune escape. The acidic environment is formed by continuous accumulation of highly acidic substances such as lactic acid and ketone bodies^71,72^. Studies report past decade have found that aerobic glycolysis and the resulting acidification of tumor microenvironment (TME) exert specific inhibitory effects on antitumor immune response mediated by T cells and the activity of tumor-infiltrating myeloid cells. Therefore, targeting sugar metabolism and/or lactic acid production and secretion is an attractive anticancer treatment strategy^73,74^. Moreover, the current study explored inflammatory infiltration landscape in HCC tissues based on 22 immune cells using the CIBERSORT tool^75^. T cells CD8 are important immunomodulatory cytokines, that play a critical role at the interface between innate and adaptive immunity, mainly in antitumor immune response^76–82^. Macrophages M2 promote tumor progression and poor prognosis, mainly promoting metastases in target organs^82–84^. Thee findings showed that enhanced glycolytic activity contributes to the highly acidic environment in the TME. Tumor-reactive T cells are suppressed resulting in loss-of-function in the acidic TME induced by glycolytic activity, resulting in a critical barrier for efficacy of cancer immunotherapy^85–87^. Most immunotherapies target the immune system but not cancer, therefore, immunotherapies are promising foundation for development of treatment regimens for several tumor types. However, complexity of the metabolic regulation of immune cell subsets and effect of the TME may have significant implications for efficacy of these therapies. In the current study, 8 genes implicated in prognosis of HCC were identified. These genes are important regulators in glucose metabolism and energy production, mainly in glycolysis process. These findings indicate that glycolysis pathway is required for proliferation of most cancer cells and for energy production in reprogramming of tumor microenvironment characteristics. Therefore, therapeutic agents can be developed that target glycolysis pathway, thus controlling tumor progression and improving patients prognosis. These findings have remarkable prognostic and therapeutic implications for HCC patients.

Notably, the current study has some limitations. First, univariate Cox regression analysis and the LASSO method were used to filter glycolytic genes associated with clinical outcomes of HCC and a prognostic model was built through multivariate Cox-PH regression analysis. In the linear regression model, adjustments were made stepwise in major groups, to reveal which variables contributed the most to confounding, and some important components with similar contributions may be ignored. Second, a prognostic risk model was developed and validated based on public databases, which was not verified by prospective clinical trials. Further studies should consider some traditionally recognized clinical factors, which have a significant effect on tumor progression and prognosis of HCC patients. Factors related to a clinical interaction may be missed, such as tumor volume, TP53 mutation, CTNNB1 mutation, lifestyle, patient follow-up time, and relevant therapeutic information. These factors have an effect on the accuracy of the prediction of the model. Therefore, the predictive performance of predictive models based on glycolysis-related gene signatures should be explored further in subsequent studies. In addition, well-designed, prospective, multicenter collaborative trials should explore if clinical decision-making based on these approaches leads to improved clinical risk stratification. To circumvent these limitations, more in vivo and in vitro studies should be conducted to verify the findings of the current study and explore more complex and in-depth biological mechanisms.

## Conclusion

The current study developed and optimized a novel 8-gene signature for identifying outcomes and recurrence in HCC patients. This predictive model improves accuracy of predicting patient prognosis. Moreover, the 8-gene signature serves as an independent prognostic factor and was superior compared with other clinicopathological features. A nomogram was established based on the GRGPI signatures and clinicopathological characteristics, which significantly improved prognosis in terms of discrimination and effectiveness of clinical decision-making. The findings of the current study provide a basis for prognostic stratification for designing prospective trials of risk-adapted therapies and surveillance strategies. In addition, the findings have clinical implications, and can be used to ensure that more patients benefit from additional systemic treatment.

## Supplementary Information

Supplementary Information.

## Data Availability

Underlying research materials can be available upon request by contacting the corresponding author.

## Abbreviations

GEO: Gene expression omnibus database
TCGA: The Cancer Genome Atlas
ICGC: International Cancer Genome Consortium
DEGs: Differentially expressed genes
AUC: Area under ROC curve
CI: Confidence interval
WGCNA: Weighted Gene Co-expression Network Analysis
PFS: Progression-free survival
MSigDB: Molecular signatures database
GSEA: Gene set enrichment analysis
t-SNE: T-distributed stochastic neighbor embedding
HBV: Viral hepatitis type B
HCV: Viral hepatitis type C
AFP: α-Fetoprotein
BCLC: Barcelona Clinic Liver Cancer
DCA: Decision curve analysis
