# Supplementary material for: Glycolysis-related gene expression profiling serves as a novel prognosis risk predictor for human hepatocellular carcinoma
Source: Sci Rep. 2021 Sep 23;11:18875. doi: 10.1038/s41598-021-98381-2 (PMC8460833; doi:10.1038/s41598-021-98381-2)
Supplement: Supplementary file 1 — Supplementary Information. [file 41598_2021_98381_MOESM1_ESM.pdf]

Glycolysis-related gene expression profiling serves as a novel prognosis risk predictor for human hepatocellular carcinoma

Lingyu Zhang[1,*], Yu Li[2,*], Yibei Dai[1], Xuchu Wang[1], Ying Cao[1], Weiwei Liu[1], Zhihua Tao[1]

[1]Department of Laboratory Medicine, The Second Affiliated Hospital of Zhejiang University School of Medicine, Hangzhou, 310009, China

[2]Department of Biochemistry and Molecular Biology, Bengbu Medical College, Anhui 233030, China

*These authors contributed equally to the work.

Correspondence to: Zhihua Tao, Department of Laboratory Medicine, The Second Affiliated Hospital of Zhejiang University School of Medicine, 88 Jiefang Road, Hangzhou, Zhejiang 310009, P.R. China, Email: zrtzh@zju.edu.cn; Weiwei Liu, Department of Laboratory Medicine, The Second Affiliated Hospital of Zhejiang University School of Medicine, 88 Jiefang Road, Hangzhou, Zhejiang 310009, P.R. China, Email: liuweiwei@zju.edu.cn.

Table S1. Core genes of 6 solid tumors.

Cancer Type	Sample numbers	Genes	Core Enrichment
**BLCA**	**Normal 19 Tumor 414**	AAAS, ACSS2, ADPGK, AGRN, AKR1A1, ALDOA, ALDOC, ALG1,ANKZF1,ARTN,AURKA,B3GALT6,B3GAT3,B3GNT3,B4GALT2,B4GALT7,BPGM, BPNT1,CASP6,CDK1,CENPA,CHPF,CHPF2, CHST1,CHST12,CHST6,CLDN9,CLN6,COG2,COPB2,DDIT4,DEPDC1,DHTKD1,EFNA3,EIF6,ENO1,ENO2,ERO1A,EXT1,EXT2,FH, FKBP4,G6PC,GALE,GALK1,GALM,GAPDH,GAPDHS,GFPT1, GLCE,GMPPA,GMPPB,GPI,GUSB,GYS1,HAX1,HK1,HK2,HK3, HMMR,HS2ST1,HS6ST2,HSPA5,IDH2,IDH3B,IDH3G,IDUA, IGFBP3,IL13RA1,KDELR3,KIF20A,LDHA,LDHAL6A,LDHC, MDH2,MED24,MIF,MIOX,MIR210,NANP,NASP,NDC1,NDUFV3, NOL3, NSDHL, NUP107,NUP155,NUP160,NUP188,NUP205, NUP210,NUP35,NUP37,NUP42,NUP43,NUP50,NUP58,NUP62, NUP85,NUP93,OGT,P4HA1,PAXIP1,PC,PCK2,PDHA1,PDK3, PFKFB1,PFKFB2,PFKFB4,PFKL,PGK1,PGLS,PGM2,PGP,PHKA2, PKM,PLOD1,PMM2,POLR3K,POM121C,PPFIA4,PPIA,PPP2R1A, PPP2R1B,PPP2R5D,PRKAG1,PSMC4,PYGM,RAE1,RARS1,RBCK1,RPE,SDHB,SDHC,SEC13,SEH1L,SLC16A3,SLC25A10,SLC25A13, SLC37A4,SOD1,SOX9, SPAG4,SRD5A3,STC2,STMN1,TGFA, TGFBI, TP53, TPI1,TPR,TREH, TSTA3,VCAN, XYLT2	**YES**
**BRAC**	**Normal 113 Tumor 1103**	AAAS,ACSS2,ADH1A,ADH1B,ADH1C,ADH4,ADH5,ADH6, ADPGK,AGRN,AKR1A1ALDH1A3,ALDH2,ALDH3A2,ALDH7A1, ALDOA,ALDOC,ALG1,ARTN,AURKA, B3GALT6, B3GAT3,B4GALT1,B4GALT2,B4GALT4, B4GALT7, BIK, BPGM, BPNT1, CACNA1H, CASP6,CBFA2T3,CDK1,CENPA, CHPF, CHPF2, CHST6, CLDN3,CLN6,COG2, COL5A1, COPB2, CXCR4, DEPDC1, DHTKD1, EFNA3, EGLN3,EIF6,ELF3,ENO1,ENO2, ERO1A, FAM162A, FBP1, FH, FKBP4, FUT8, GALE,GALK1, GAPDH, GMPPA, GMPPB, GNPDA1, GOT2,GPC1, GPC4, GPI, HAX1, HK1,HMMR,HSPA5,IDH2,IDH3G, IDUA, IER3, ISG20, JMJD8,KDELR3, KIF20A,LDHA,LDHB,MDH2, ME2, MED24, MIF,MIOX, MIR210,NASP,NDC1, NSDHL, NUP107, NUP133, NUP155, NUP188, NUP205, NUP210, NUP37, NUP43, NUP50, NUP58, NUP62,NUP85, NUP93,OGDHL,P4HA1,P4HA2, PAXIP1, PCK1, PCK2,PDK3,PFKFB1, PFKFB3,PFKFB4,PFKL, PFKP, PGAM1, PGK1,PGK2, PGLS, PGM1,PGM2, PGM2L1, PGP, PKM, PLOD1, PMM2, POLR3K, PPIA, PPP2CA, PPP2CB, PPP2R1A, PPP2R1B,PPP2R5D, PRKAG1, PRPS1, PSMC4QSOX1, RAE1, RARS1,RBCK1, RPE,SAP30,SDC1,SDHC,SEC13,SEH1L,SLC16A3, SLC25A10, SLC25A13, SLC35A3,SLC37A4,SOD1,SPAG4,SRD5A3,	**YES**

		STMN1, TALDO1,TFF3,TIGAR,TP53,TPBG,TPI1,TPR,TSTA3,TXN, VCAN, VEGFA,XYLT2, ZBTB7A	
**HNSC**	**Normal 44 Tumor 502**	AAAS,ABCB6,AC016586.1,ACSS2,ACTN3, ADH1A, ADH1B, ADH4,ADH6, ADH7,ADPGK,AGRN,AKR1A1, ALDH1A3, ALDH2, ALDH3A1, ALDH3A2, ALDH3B1, ALDH3B2, ALDH7A1, ALDH9A1, ALDOA,ALDOC, ALG1, ANKZF1, ARPP19, ARTN, AURKA, B3GALT6, B3GAT3,B4GALT1,B4GALT2, B4GALT7, BPGM, CACNA1H,CASP6,CD44, CDK1, CENPA,CHPF, CHPF2, CHST1, CHST12, CHST2,CLN6, COL5A1,COPB2, CXCR4, DDIT4, DEPDC,1 DLAT, DLDEGFR, EGLN3,ENO1, ENO2,ENO3,EXT1, EXT2, FBP2, FKBP4, G6PC2, G6PD, GALK2,GAPDH, GCK, GMPPA, GNPDA1, GPC1, GPI, GPR87, HIF1A,HK1, HK2, HK3, HKDC1, HMMR, HS2ST1, HSPA5, IGFBP3, INS, ISG20, KDELR3, KIF20A, LDHA, MET, MIF, MIOX, NANP, NASP, NDC1, NT5E, NUP107, NUP133, NUP153, NUP155, NUP160, NUP188, NUP205, NUP210, NUP214, NUP35, NUP37, NUP42, NUP43, NUP50, NUP54, NUP58, NUP62, NUP85, NUP93, OGT, P4HA1, P4HA2, PC, PDHA1, PDHB, PDK3, PFKFB1, PFKFB3, PFKFB4, PFKL, PFKM, PFKP, PGAM1, PGK1, PGM1,PGM2, PGP, PKLR, PKM, PLOD1, PLOD,2POLR3K, POM121, POM121C, PPIA, PPP2CA, PPP2CB, PPP2R1A, PPP2R5D, PRKACA, PRKACB, PRPS1, PRXL2C, PSMC4, PYGL, PYGM, RAE1, RBCK1, RPE, SAP30, SEH1L, SLC16A3, SLC25A12, SLC25A13, SPAG4, STC2, STMN1, TGFA, TGFBI, TIGAR, TPBG, TPI1, TPR, TPST1, TREH, VCAN, VEGFA, XYLT2	**YES**
**LIHC**	**Normal 50 Tumor 374**	AAAS,ABCB6,AC016586.1,ACO2,ADH1A, ADH1B, ADH1C, ADH4,ADH6, ADPGK, AGRN, ALDH1B1, ALDH2, ALDH9A1, ALDOA, ALDOB, ALG1, ANKZF1, ARNT, ARPP19, ARTN, AURKA, B3GALT6, B3GAT3, B3GNT3, B4GALT2, B4GALT4, B4GALT7, BIK, BPGM, BPNT1, CDK1,CENPA, CHPF2, CHST1, CHST12, CHST6, CLN6, COG2, COL5A1, COPB2, DDIT4, DEPDC1, DPYSL4, ECDEFNA3, EGLN3, EIF6, ENO1, ENO3, ERO1A, EXT2, FBP1, FKBP4, G6PC, G6PD, GAL3ST1, GALE, GALK1,GALK2,GAPDH,GFPT1,GLCE,GMPPA,GMPPB,GNPDA1, GNPDA2,GPC3, GPI, GYS1, HAX1, HDAC4,HDLBP, HK2, HK3, HKDC1, HMMR, HOMER1, HS2ST1, HSPA5, IDH3B, IDH3G,IDUA, KDELR3, KHK, KIF20A, KIF2A, MDH1, MDH2, ME1, ME2, MED24, MET, MIF, MIOX, MIR210, MLXIPL, MPI,NANP, NASP, NDC1, NDUFV3, NOL3, NSDHL, NUP107, NUP133, NUP153, NUP155, NUP160, NUP188, NUP205, NUP210, NUP214, NUP35, NUP37, NUP42, NUP43, NUP62, NUP85, NUP93, OGDH, OGT P4HA2, PAM,PAXIP1, PCK1, PCK2, PFKFB2, PFKFB4, PFKL, PFKM, PGK1, PGLS, PGM1, PGP,	**YES**

		PHKA2,PKM,PLOD1, POLR3K, POM121,POM121C,PPFIA4, PPIA, PPP2CA, PPP2R1A, PPP2R5D, PRKAA2, PRKAG1, PRPS1, PSMC4, PYGB, RAE1, RARS1, RBCK1, RPE, RRAGD, SAP30, SDC2, SDHC, SEC13, SLC16A3, SLC25A10, SLC25A12, SOX9,SPAG4, SRD5A3, STC1, STC2, STMN1, TALDO1, TGFBI, TIGAR, TP53, TPI1, TPR, TREH, TSTA3, TXN, VCAN,VEGFA,XYLT2, ZBTB7A, ZNF292	
**LUAD**	**Normal 59 Tumor 533**	AAAS, ABCB6,ADORA2B,AGL,AGRN,AK4, AKR1A1,ALDH1B1, ALDH3B2, ALDH7A1, ALDOA, ALDOC,ALG1, ANKZF1,ARTN, AURKA, B3GALT6, B3GAT1,B3GAT3, B3GNT3, B4GALT1, B4GALT2, B4GALT4,B4GALT7,BIK,BPGM,BPNT1, CAPN5, CASP6, CDK1,CENPA, CHPF, CHPF2,CLDN3, CLDN9, COG2,COL5A1,COPB2, CTH,DDIT4,DEPDC1,DHTKD1, DLAT,DLDDSC2,ECD, EFNA3,EGLN3,EIF6, ELF3, ENO1,ENO2, ENO3, ENTPD5, ERO1A,FAM162A, FH, FKBP4,FUT8,G6PD, GAL3ST1, GALE, GALK1, GALM,GAPDH, GAPDHS, GCKR,GCLC, GFPT1, GLCE,GMPPA, GMPPB,GNPDA1,GOT1, GOT2,GPC1, GPI,GPR87, GUSB, GYS1, HAX1,HDLBP, HIF1A,HKDC1,HMMR,HS2ST1, HS6ST2, HSPA5, IDH1, IDH2, IDH3B, IDH3G, IDUA, IER3, IGFBP3, ISG20, KDELR3, KHK,KIF20A, KIF2A, LCT, LDHA, LDHB, LHPP, MDH1,MDH2, ME1, ME2, MET, MIF, MIOX, MIR210,MPI, NANP, NASP, NDC1, NDUFV3,NOL3, NSDHL,NT5E, NUP107, NUP133, NUP153, NUP155, NUP160,NUP188, NUP205, NUP210, NUP35, NUP37, NUP42, NUP43,NUP50, NUP54, NUP62, NUP85, NUP88, NUP93,OGTP4HA1, PAM, PAXIP1, PC, PDHA1, PDHB, PDK3,PFKFB1, PFKFB4, PFKL, PFKP, PGAM1, PGAM4, PGK1,PGK2, PGLS, PGM2, PGM2L1, PGP, PHKA2, PKM,PKP2,PLOD1,PLOD2, PMM2, POLR3K, POM121, POM121C,PPARA,PPFIA4,PPIA,PPP2R1A,PPP2R1B,PPP2R5D,PRKAA1, PRKAA2, PRKAG1, PRPS1, PSMC4, PYGB, PYGL,QSOX1, RAE1,RRS1, RBCK1, RPE, SAP30, SDC1,SDHA, SDHB, SDHC,SEC13, SEH1L, SLC16A3,SLC25A10, SLC25A12, SLC25A13, SLC35A3,SLC37A4,SOD1, SOX9, SPAG4, SRD5A3, STMN1, SUCLG1,TALDO1,TGFA, TIGAR, TP53, TPBG, TPI1, TPR,TPST1, TSTA3, TXN,UGP2, VCAN, VEGFA, XYLT2,ZNF292	**YES**
**LUSC**	**Normal 49 Tumor 502**	AAAS, ABCB6,ADH7,ADORA2B, AK4,ALDH3A1, ALDH3B2, ALDOA, ALDOC, ANKZF1, ARTN, AURKA, B3GAT3, B3GNT3, B4GALT2, B4GALT4, B4GALT7, BIK, CASP6, CDK1, CENPA, CHPF, CHPF2, CHST2, COL5A1, COPB2, DDIT4, DEPDC1, DHTKD1, DLD, DPYSL4, DSC2, EFNA3, EGFR, EGLN3, EIF6, ENO1, ENO2, ERO1A, EXT1,FAM162A, FH, FKBP4, FUT8,	**YES**

		G6PD, GAPDH, GCLC, GMPPA, GNPDA1, GOT1, GOT2, GPC1, GPI, GPR87, GYS1, HAX1, HIF1A, HK2, HMMR, HS6ST2, HSPA5, IDH1, IDH2, IGFBP3, KDELR3, KIF20A, KIF2A, LDHA, LDHB, LDHC, MDH1, MDH2, ME1, MIF, MIOX, MIR210, MPI, NANP, NASP, NDC1, NOL3, NSDHL, NUP107, NUP155, NUP205, NUP210, NUP35, NUP37, NUP42, NUP43, NUP50, NUP62, NUP85, NUP88, NUP93, OGDHL, P4HA1, PAXIP1, PC, PDK3, PFKFB1, PFKFB2, PFKFB3, PFKFB4, PFKP, PGAM1, PGAM4, PGK1, PGK2, PGM1, PGM2, PGM2L1, PKM, PKP2, PLOD1, PLOD2, PMM2, POLR3K, PPFIA4, PPIA, PPP2CA, PPP2CB, PRKACA, PRKACB, PRKACG, PSMC4, PYGL, PYGM, RAE1, RARS1, RPE, SAP30, SDC1, SDHA, SDHC, SEH1L, SLC16A3, SLC25A10, SLC25A12, SLC25A13, SLC37A4, SOD1, SOX9, SPAG4, SRD5A3, STC2, STMN1, TALDO1, TGFA, TP53, TPBG, TPI1, TPST1, TREH, TSTA3, TXN, VCAN, XYLT2	

Table S2. Multivariable Cox regression analysis of predictors of survival outcomes in the TCGA discovery cohort for 6 solid tumors.

Cancer type	Gene	Hazzard Ratio	β(Cox)	*P* value
	NUP188	1.598825	0.058158	0.002597
**BLCA**	CHPF	1.551001	0.549488	0.002997
	AK3	0.969007	-0.314831	0.002906
	PGK1	1.266736	0.665145	0.001651
**BRAC**	SDC1	1.162645	0.162513	9.40E-05
	CHPF	1.490339	0.048914	0.000140
	NUP43	1.469928	0.059221	4.43E-05
	ALDH1B1	1.229825	0.227224	0.004188
	STC2	1.303147	0.298643	9.03E-06
**HNSC**	ALDH2	0.985525	-0.145801	0.005879
	PGK1	1.247946	0.247639	4.03E-05
	PYGL	1.463654	0.462582	0.013119
	GPR87	1.585657	0.583949	0.004114
	KIF20A	1.128769	0.242487	1.37E-09
	AURKA	1.019880	0.012238	0.000375
	HMMR	1.121071	0.114007	1.01E-05
**LIHC**	STMN1	1.021461	0.091125	1.46E-05
	DEPDC1	1.229999	0.132254	8.40E-06
	PFKFB4	1.252219	0.156219	1.69E-05
	CENPA	1. 176523	0.092026	1.40E-07
	CDK1	1.052063	0.053426	1.25E-06
	PPP2R1A	1.116637	0.115962	4.26E-11
	RBCK1	1.104909	0.104363	0.010349
	SLC16A3	1.103470	0.102935	8.73E-05

**LUAD**	FKBP4	1.814383	0.281108	4.50E-08
	ERO1A	1.547494	0.546001	1.67E-06
	PPIA	1.476700	0.475567	0.021940
	B4GALT1	1.353855	0.353231	0.013259
	LDHA	1.295509	0.295073	9.01E-07
	PFKM	1.333626	0.133353	0.007008
**LUSC**	HMMR	1.431428	0.314246	0.018149
	TPST1	1.14307	0.143068	0.000486

Table S3. Clinicopathological characteristics of the HCC patients enrolled in the TCGA cohort.

**Clinicopathological Characteristics**	**Risk Score (OS)**		χ^2	***P* value**	**Risk Score (RFS)**		χ^2	***P* value**
	**High Risk n (%)**	**Low Risk n (%)**			**High Risk n (%)**	**Low Risk n (%)**		
**Gender**			0.08769	0.7671			0.0215	0.8832
Male	126(50.40%)	124(49.60%)			124(49.60%)	126(50.40%)		
Female	59(48.76%)	62(51.24%)			61(50.41%)	60(49.59%)		
**Age**			2.818	0.0932			1.560	0.2116
≤65	124(53.22%)	109(46.78%)			122(52.36%)	111(47.64%)		
>65	61(44.20%)	77(55.80%)			63(45.65%)	75(54.35%)		
**T**			13.77	0.0032			11.46	0.0095
T1	76(42.70%)	102(57.30%)			76(41.30%)	108(58.70%)		
T2	48(48.00%)	52(52.00%)			55(58.51%)	39(41.49%)		
T3	51(63.75%)	29(36.25)			45(56.25%)	35(43.75%)		
T4	10(76.92%)	3(23.08%)			9(69.23%)	4(30.77%)		
**Stage**			42.73	<0.001			13.74	0.0033
I	66(37.93%)	108(62.07%)			78(41.71%)	109(58.29%)		
II	42(42.86%)	56(57.14%)			48(55.17%)	39(44.83%)		
III	68(78.16%)	19(21.84%)			51(57.96%)	37(42.05%)		
IV	9(75.00%)	3(25.00%)			8(88.89%)	1(11.11%)		
**Grade**			10.12	0.0175			2.965	0.0851
G1	16(35.56%)	29(64.44%)			40(56.34%)	31(43.66%)		
G2	77(47.83%)	84(52.17%)			43(31.85%)	92(68.15%)		
G3	80(53.33%)	70(46.67%)			91(64.54%)	50(35.46%)		
G4	12(80.00%)	3(20.00%)			11(45.83%)	13(54.17%)		
**Family Cancer History**			0.00129	0.0359			0.1665	0.6832
NO	125(49.80%)	126(50.20%)			127(50.60%)	124(49.40%)		
YES	60(50.00%)	60(50.00%)			58(48.33%)	62(51.67%)		
**Hepatitis Virus Infection**			2.481	0.4787			2.104	0.5511
None Risk	91(46.67%)	104(53.33%)			103(52.82%)	92(47.18%)		
HBV	32(52.46%)	29(47.54%)			31(50.82%)	30(49.18%)		
HCV	8(44.44%)	10(55.56%)			8(44.44%)	10(55.56%)		
HCV&HBV	54(55.67%)	43(44.43%)			43(44.33%)	54(55.67%)		

**Child Pugh Score**			5.206	0.0741			3.480	0.1755
A	88(44.44%)	110(55.56%)			90(45.46%)	108(54.54%)		
B	54(54.55%)	45(45.45%)			53(53.54%)	46(46.46%)		
C	43(58.11%)	31(41.89%)			42(56.76%)	32(43.24%)		
**BMI**			1.683	0.1946				
<=24	93(53.45%)	81(46.55%)			93(53.45%)	81(46.55%)		
>24	92(46.70%)	105(53.30%)			92(46.70%)	105(53.30%)		
**AFP**			5.952	0.0147			1.424	0.2328
<=200	83(43.68%)	107(56.32%)			89(46.84%)	101(53.18%)		
>200	102(56.35%)	79(43.65%)			96(53.04%)	85(46.96%)		
**Cancer Status**			16.64	<0.001				
Tumor Free	98(41.88%)	136(58.12%)						
With Tumor	72(65.45%)	38(34.55%)						
**Recurrence Event**			76.91	<0.001				
NO	82(42.93%)	109(57.07%)						
YES	103(57.22%)	77(42.78%)						
**Status**								
Alive	90(37.34%)	151(62.66%)	43.13	<0.001				
Dead	95(73.08)	35(26.92)						

Table S4. Clinicopathological characteristics of the HCC patients enrolled in the GEO cohort.

**Clinicopathological Characteristics**	**Risk Score (OS)**		χ^2	***P* value**
	**High Risk n (%)**	**Low Risk n (%)**		
**Gender**			2.553	0.1101
Male	91(47.64%)	100(52.36%)		
Female	19(63.33%)	11(36.67%)		
**Age**			0.0430	0.8355
≤65	100(50.00%)	100(50.00%)		
>65	10(47.62%)	11(52.38%)		
**Stage**			39.37	<0.001
I	26(27.96%)	67(72.04%)		
II	43(55.13%)	35(44.87%)		
III	41(82.00%)	9(18.00%)		
**BCLC Staging**			28.23	<0.001
Zero	9(42.86%)	12(57.14%)		
A	59(39.60%)	90(60.40%)		
B	18(81.82%)	4(18.18%)		
C	24(82.76%)	5(17.24%)		
**Child Pugh Score**			26.43	<0.001
A	32(32.99%)	65(67.01%)		
B	40(53.33%)	35(46.67%)		
C	38(77.55%)	11(22.45%)		
**Cirrhosis**			3.792	0.0515

NO	5(27.78%)	13(72.22%)		
YES	105(51.72%)	98(48.28%)		
**Multi Nodular**			8.259	0.0041
NO	79(44.89%)	97(55.11%)		
YES	31(68.89%)	14(31.11%)		
Tumor Size			12.54	<0.001
<=5	57(40.71%)	83(59.29%)		
>5	53(65.43%)	28(34.57%)		
**AFP**			14.79	<0.001
<=300	46(38.02%)	75(61.98%)		
>300	64(64.00%)	36(36.00%)		
ALT			1.655	0.1983
<=50	60(46.15%)	70(53.85%)		
>50	50(54.95%)	41(45.05%)		
**Recurrence Event**			23.08	<0.001
NO	32(32.00%)	68(68.00%)		
YES	78(64.46%)	43(35.54%)		
**Status**			54.48	<0.001
Alive	41(30.15%)	95(69.85%)		
Dead	69(81.18%)	16(18.82%)		

Table S5. Clinicopathological characteristics of the HCC patients enrolled in the ICGC cohort.

**Clinicopathological Characteristics**	**Risk Score (OS)**		χ^2	***P* value**
	**High Risk n (%)**	**Low Risk n (%)**		
**Gender**			2.691	0.1009
Male	80(46.78%)	91(53.22%)		
Female	36(59.02%)	25(40.98%)		
**Age**			2.074	0.1498
≤65	35(42.17%)	48(57.83%)		
>65	74(52.11%)	68(47.89%)		
**Stage**			10.98	0.0118
I	17(47.22%)	19(52.78%)		
II	46(43.40%)	60(56.60%)		
III	37(52.11%)	34(47.89%)		
IV	16(84.21%)	3(15.79%)		
**Grade**			28.20	<0.001
G1	6(27.27%)	16(72.78%)		
G2	56(41.55%)	83(58.45%)		
G3	42(71.19%)	17(28.81%)		
G4	9(100.00%)	0(0.00%)		
**Family Cancer History**			0.6868	0.4072
NO	79(51.97%)	73(48.03%)		

YES	37(46.25%)	43(53.75%)		
**Prior Malignancy**			0.6125	0.4338
NO	99(49.01%)	103(50.99%)		
YES	17(56.67%)	13(43.33%)		
**Status**			27.43	<0.001
Alive	79(41.80%)	110(58.20%)		
Dead	37(86.05%)	6(13.95%)		

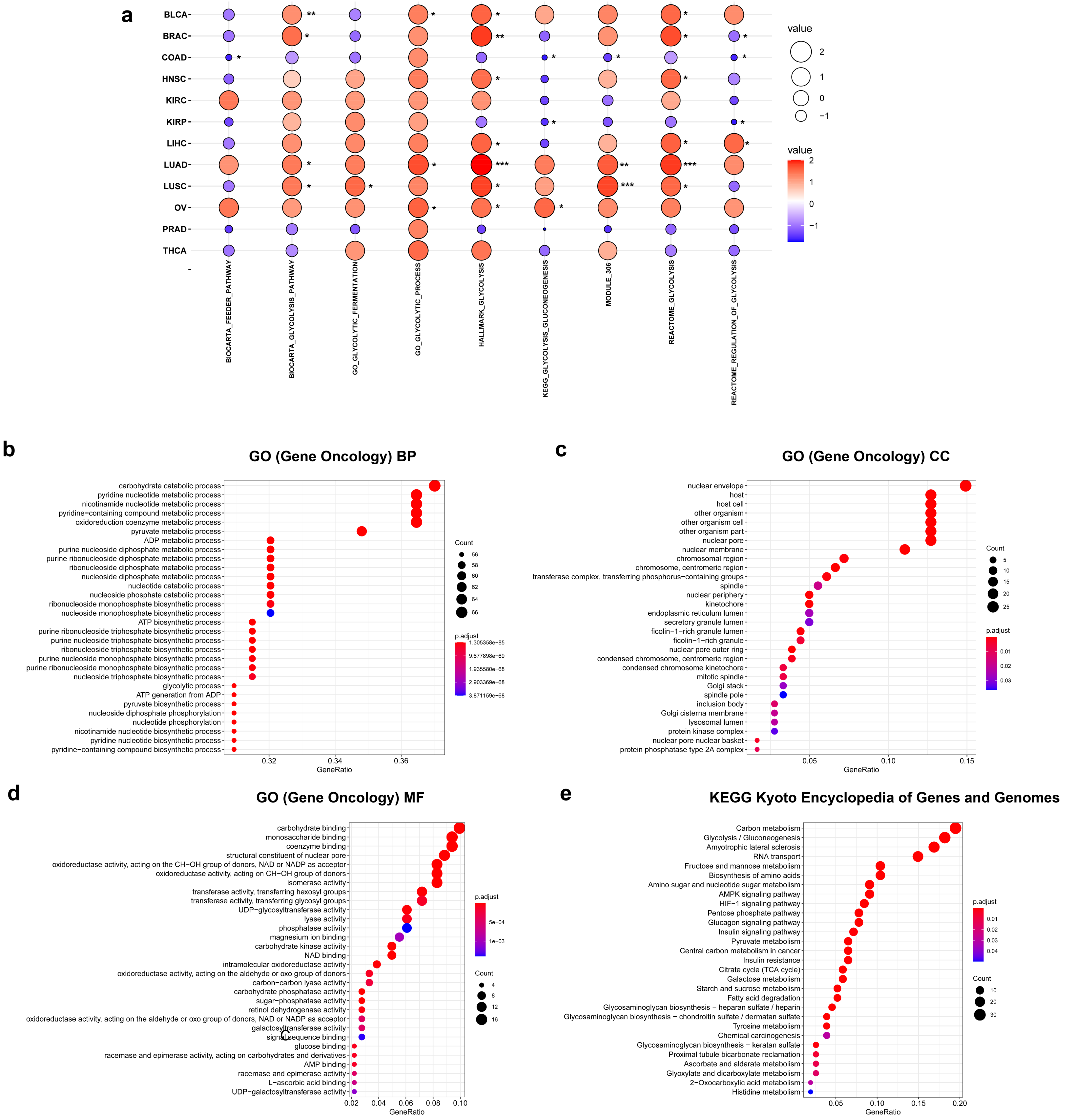

Figure S1. a Distribution of NES value and FDR q value in GSEA analysis results. (**b-e**) Gene Ontology (GO) enrichment analysis and KEGG pathways enriched core genes form GSEA results.

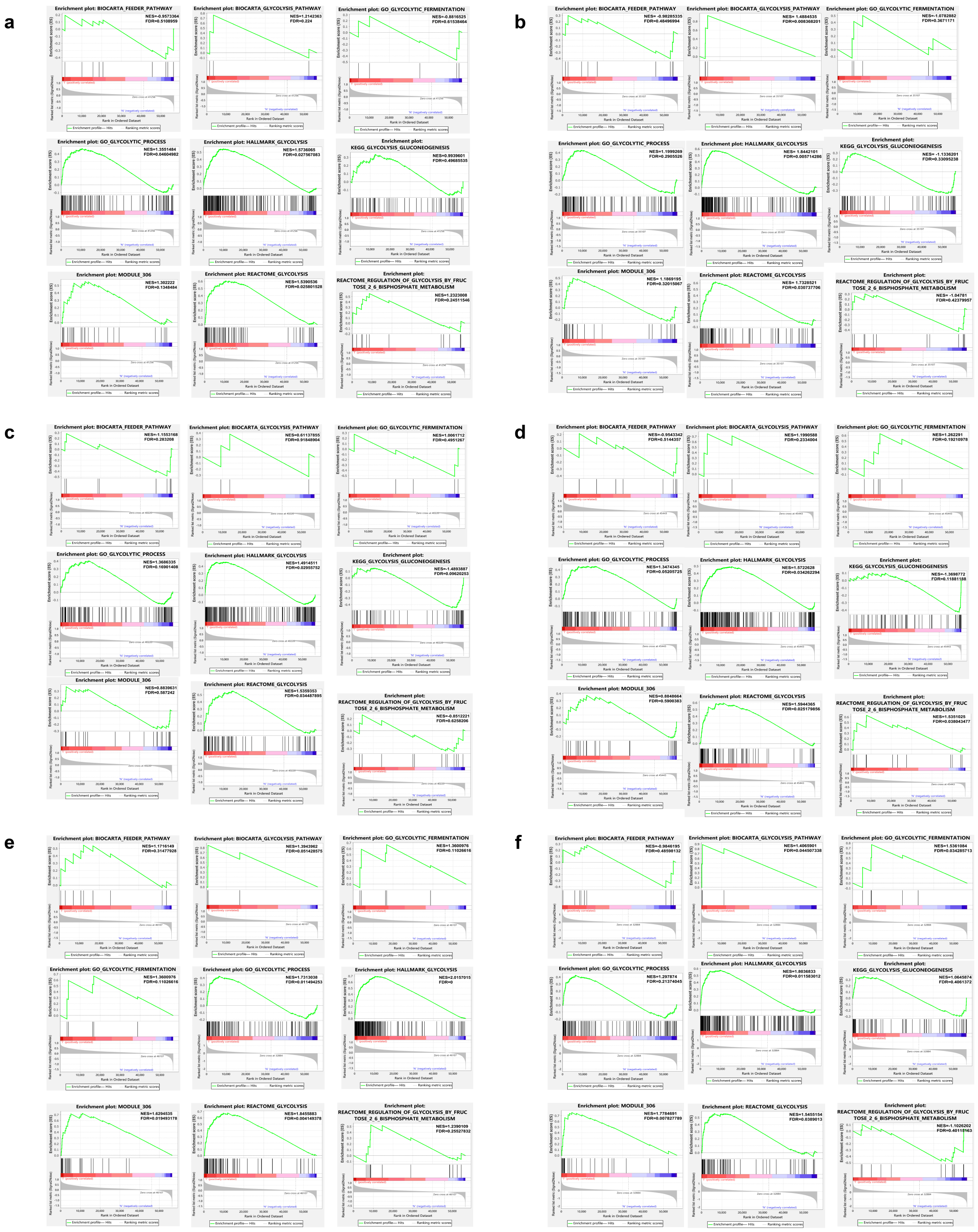

Figure S2. Detailed GSEA analyses in 6 solid tumors.

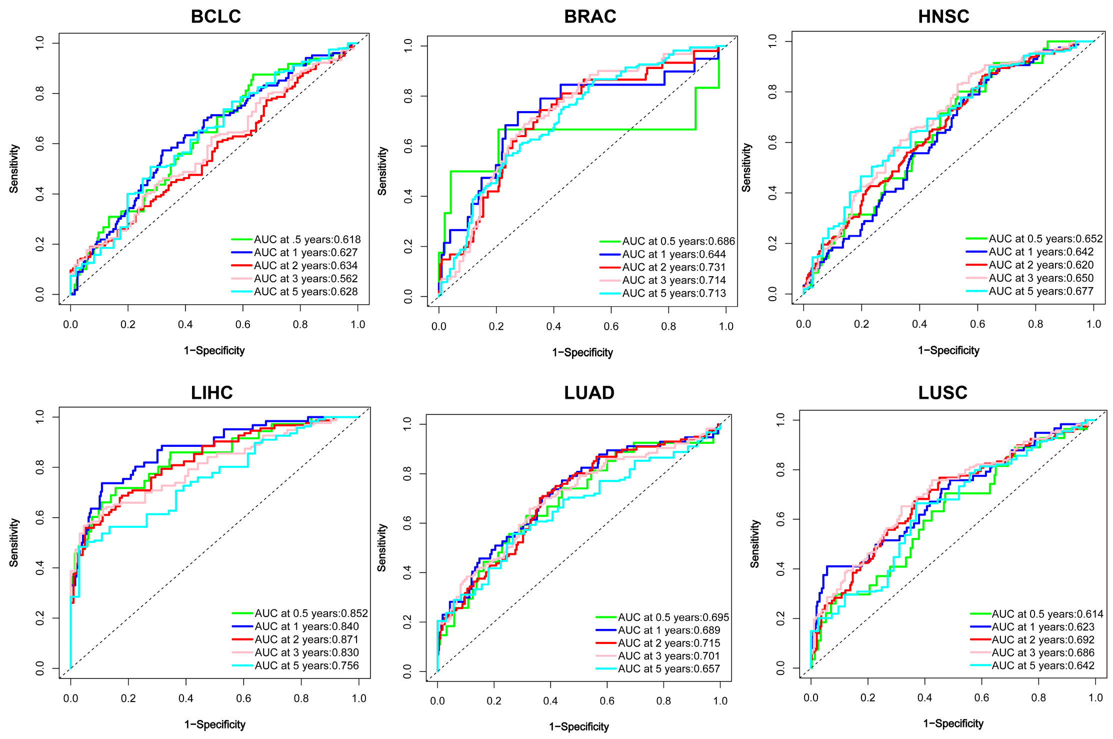

Figure S3. Performance of ROC curves in BLCA, BRCA, HNSC, LIHC, LUAD, and LUSC.

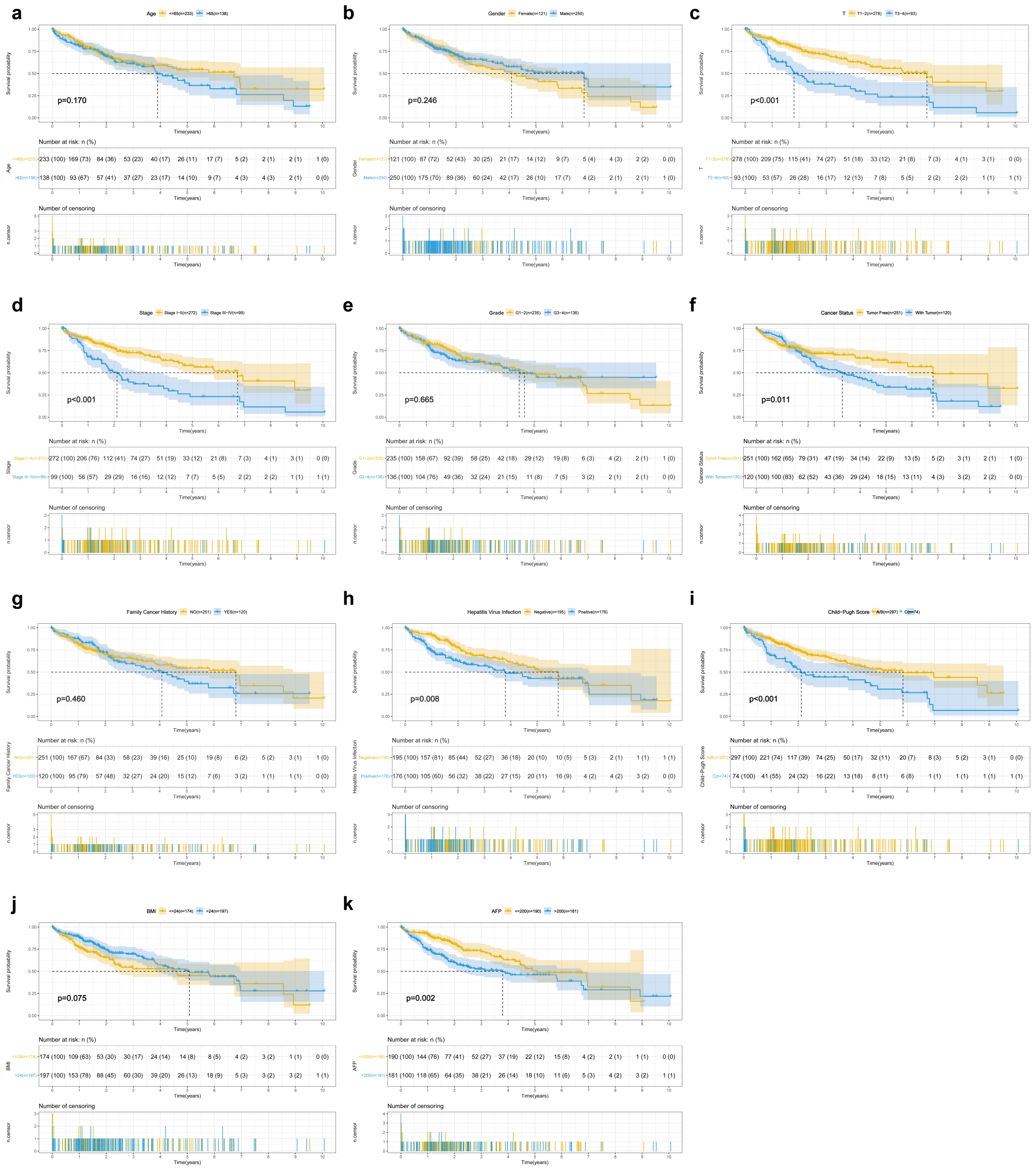

Figure S4. K-M survival analysis in different clinicopathological subgroups in the TCGA cohort.

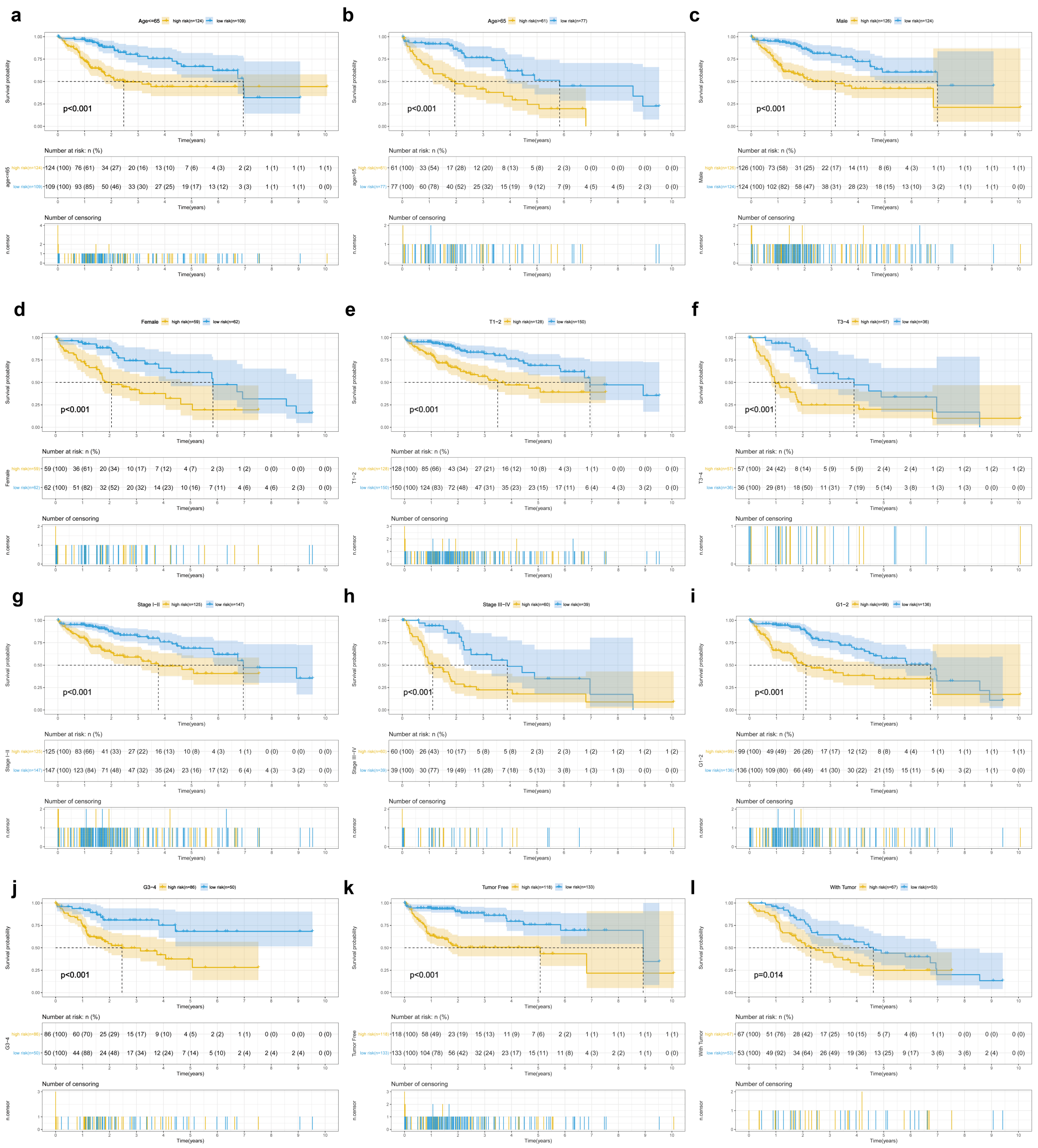

Figure S5. K-M analysis of patients stratified by age (**a, b**), gender (**c, d**), T classification (**e, f**), stage (**g, h**), grade (**i, j**), and tumor status (**k, l**) in TCGA cohort. Patients in the high-risk group achieved shorter overall survival.

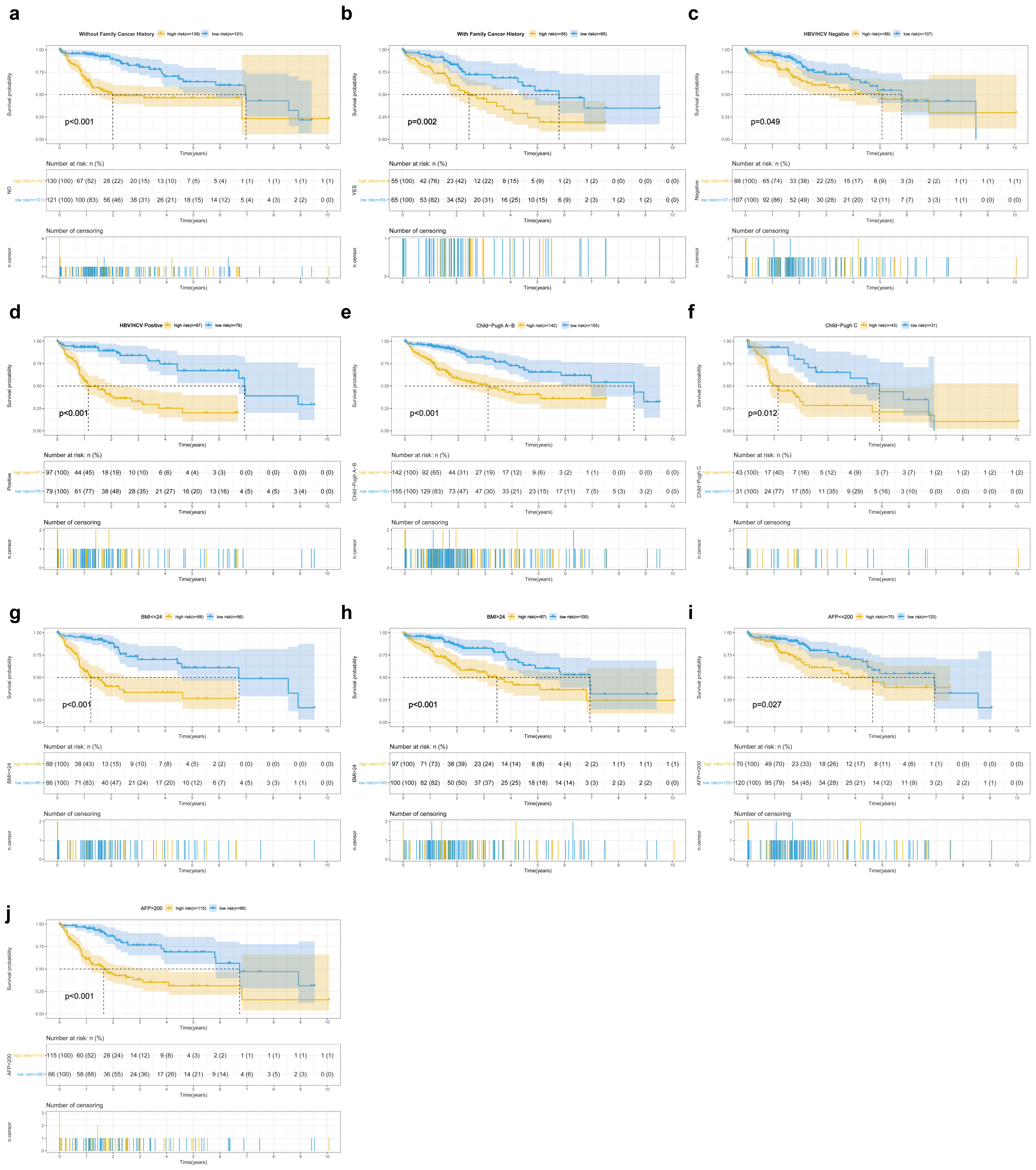

Figure S6. K-M analysis of patients stratified by family cancer history (**a, b**), hepatitis virus infection (**c, d**), Child-Pugh score (**e, f**), BMI (**g, h**), and AFP level (**i, j**) in TCGA cohort. Patients in the high-risk group achieved shorter overall survival.

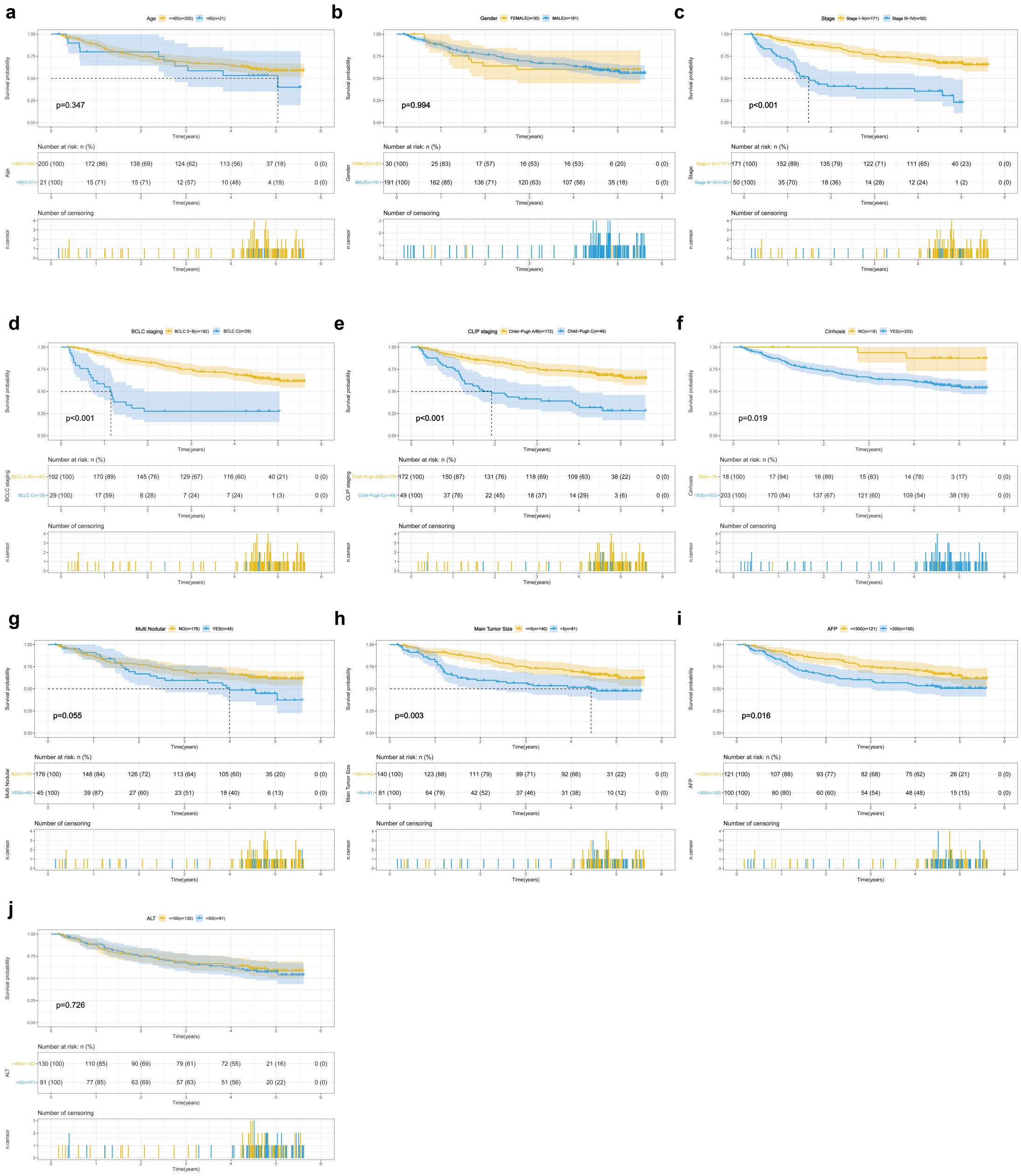

Figure S7. K-M survival analysis in different clinicopathological subgroups in the GEO cohort.

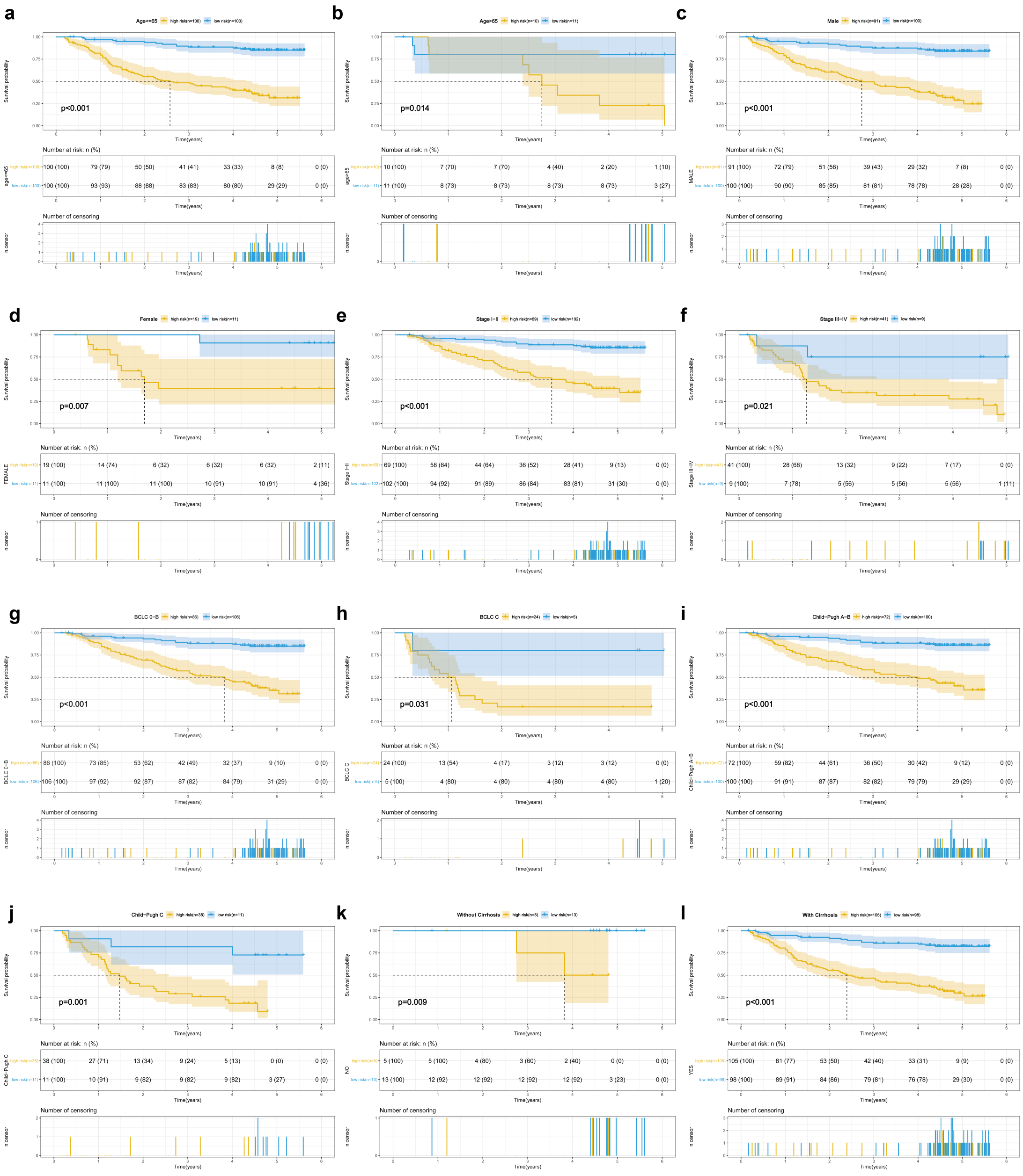

Figure S8. K-M analysis of patients stratified by Age (**a, b**), Gender (**c, d**), Stage (**e, f**), BCLC Staging (**g, h**), Child-Pugh Score (**i, j**), and Cirrhosis status (**k, l**) in GEO cohort. Patients in the high-risk group achieved shorter overall survival.

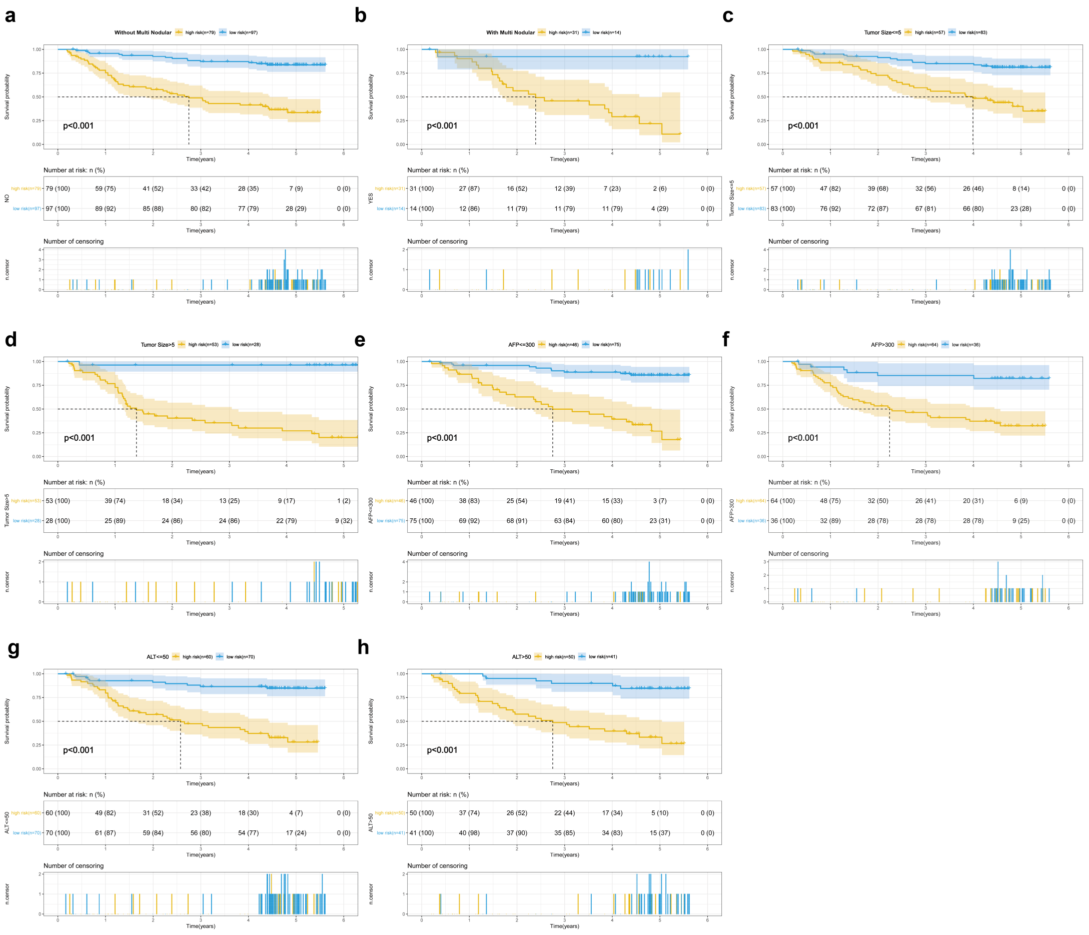

Figure S9. K-M analysis of patients stratified by Muti Nodular status (**a, b**), Tumor Size (**c, d**), AFP level (**e, f**), and ALT level (**g, h**) in GEO cohort. Patients in the high-risk group achieved shorter overall survival.

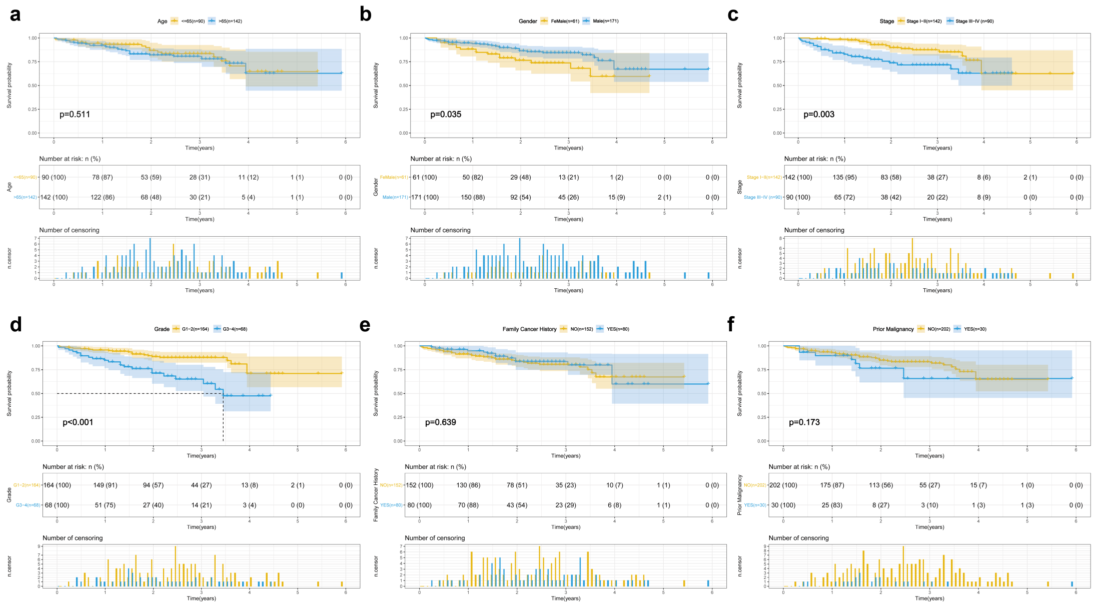

Figure S10. K-M survival analysis in different clinicopathological subgroups in the ICGC cohort.

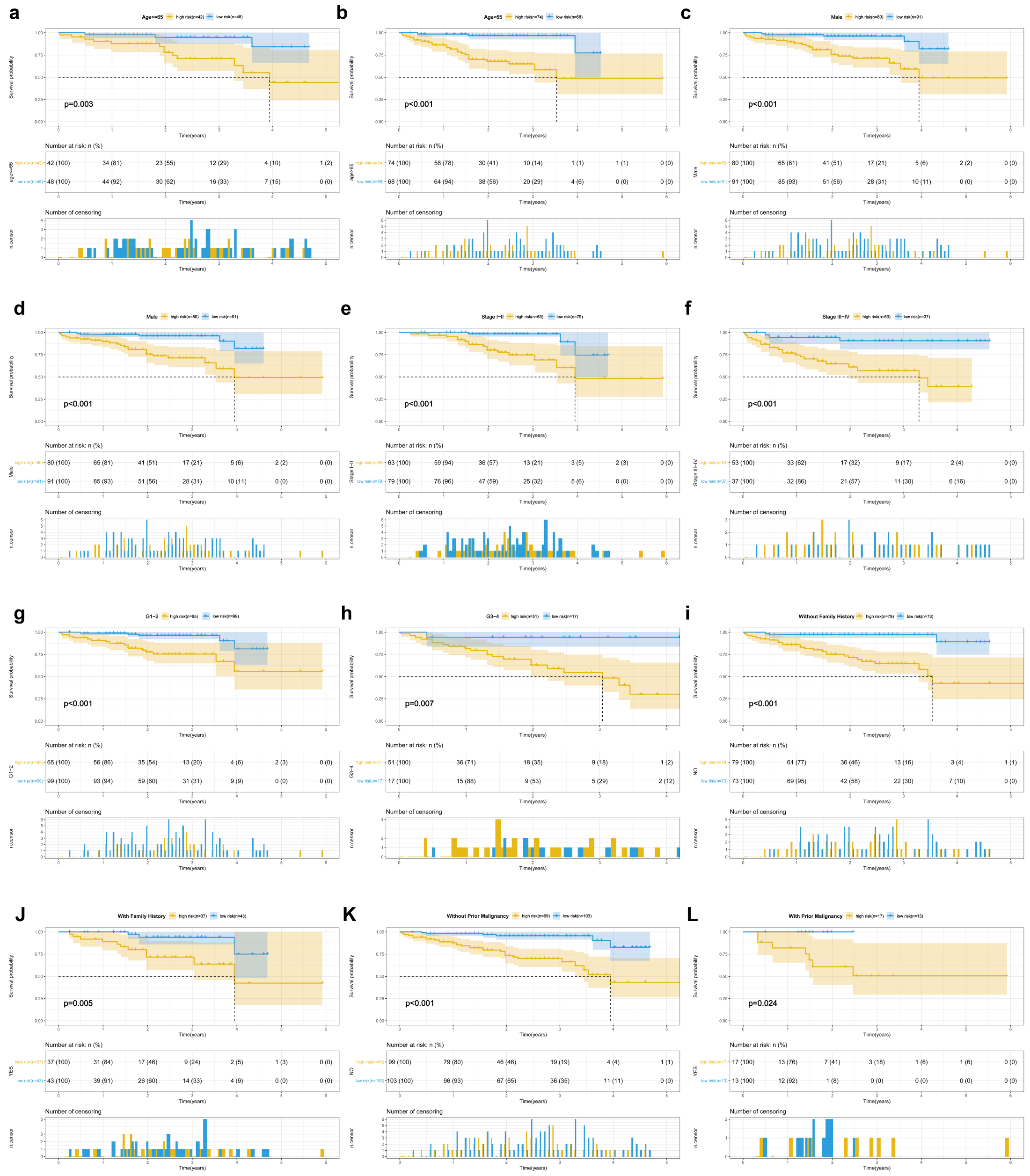

Figure S11. K-M analysis of patients stratified by Age (**a, b**), Gender (**c, d**), Stage (**e, f**), Grade (**g, h**), Family Cancer History (**i, j**), and Prior Malignancy (**k, l**) in GEO cohort. Patients in the high-risk group achieved shorter overall survival.

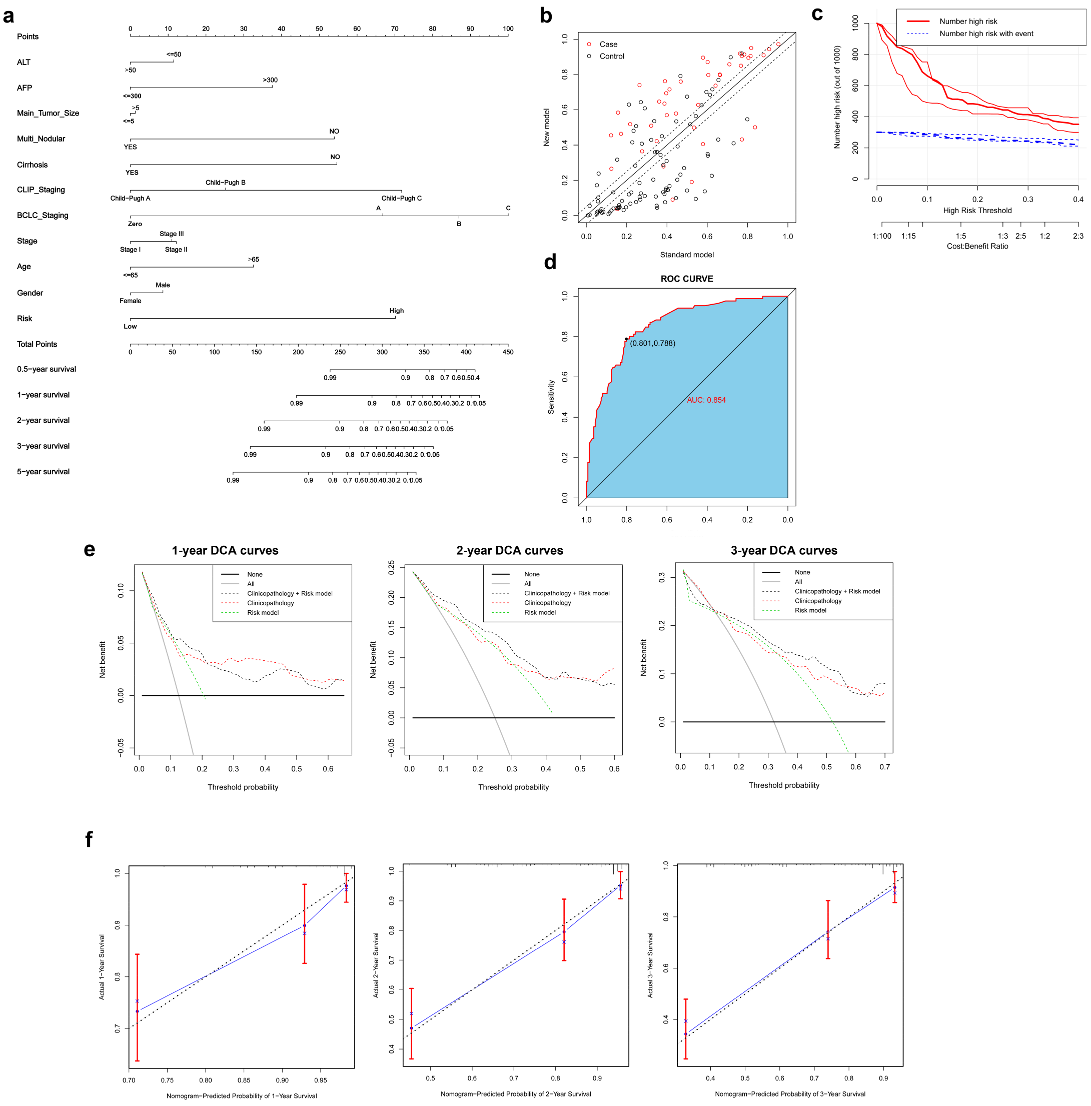

Figure S12. Performance of a nomogram integrated the GRGPI model and clinicopathological characteristics in the GEO cohort. A Nomograph for predicting overall survival probability of HCC patients. (**b-c**) The Nomogram model improves the identification of high-risk patients, and 221 HCC patients are reclassified between the standard model and the Nomogram model. **d** ROC curve for Nomogram model. (**e, f**) Decision curve and calibration curves analyses of Nomogram for 1-, 2-, and 3-year.

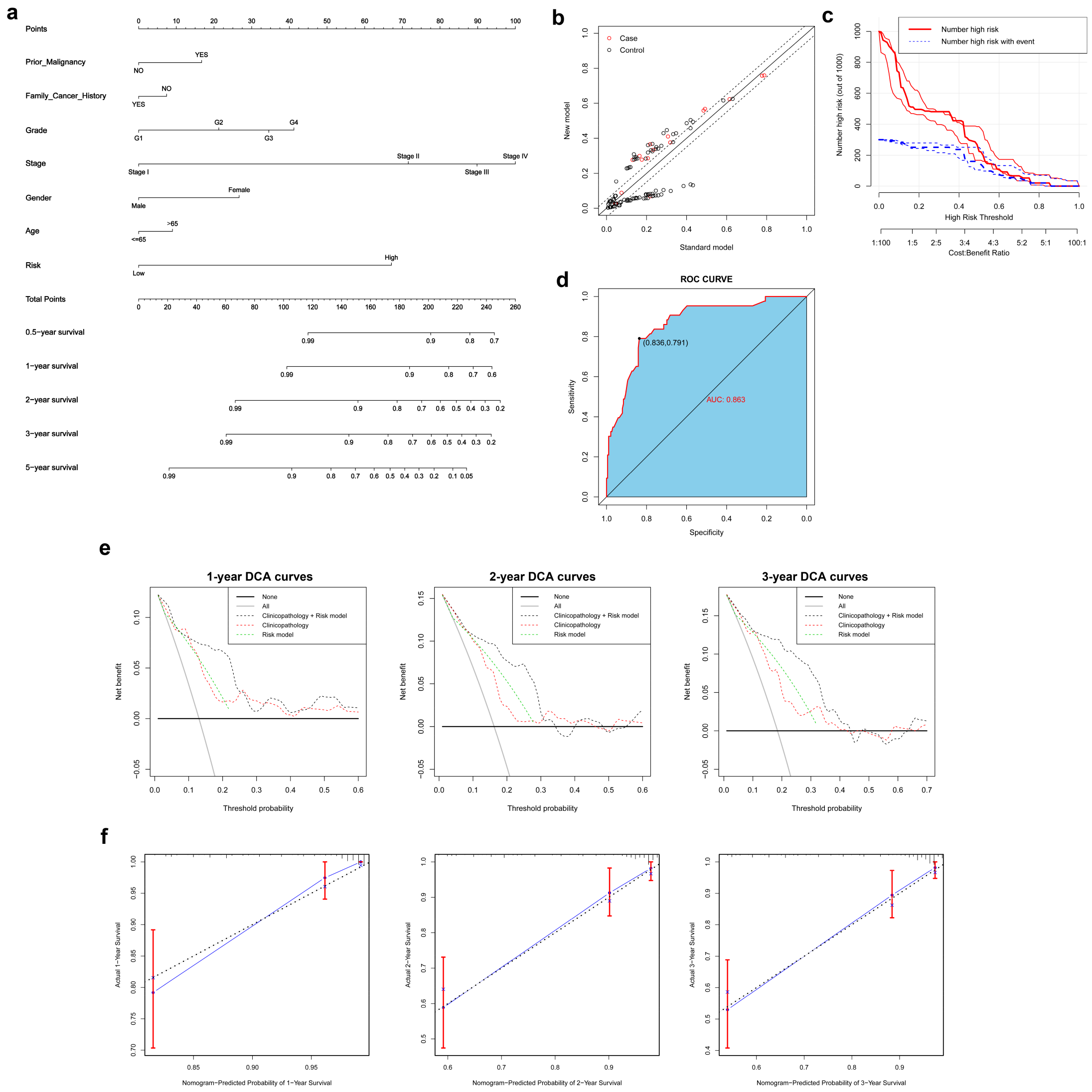

Figure S13. Performance of a nomogram integrated the GRGPI model and clinicopathological characteristics in the ICGC cohort. A Nomograph for predicting overall survival probability of HCC patients. (**b-c**) The Nomogram model improves the identification of high-risk patients, and 221 HCC patients are reclassified between the standard model and the Nomogram model. **d** ROC curve for Nomogram model. (**e, f**) Decision curve and calibration curves analyses of Nomogram for 1-, 2-, and 3-year.
